# Spatiotemporal transitions in *Pseudo-nitzschia* species assemblages and domoic acid along the Alaska coast

**DOI:** 10.1371/journal.pone.0282794

**Published:** 2023-03-22

**Authors:** Katherine A. Hubbard, Maria Célia Villac, Christina Chadwick, Alexandra A. DeSmidt, Leanne Flewelling, April Granholm, Molly Joseph, Taylor Wood, Evangeline Fachon, Michael L. Brosnahan, Mindy Richlen, Mrunmayee Pathare, Dean Stockwell, Peigen Lin, Josée N. Bouchard, Robert Pickart, Donald M. Anderson

**Affiliations:** 1 Florida Fish and Wildlife Conservation Commission-Fish and Wildlife Research Institute, Saint Petersburg, Florida, United States of America; 2 Woods Hole Oceanographic Institution, Woods Hole, Massachusetts, United States of America; 3 Department of Earth Atmospheric and Planetary Sciences, Massachusetts Institute of Technology, Cambridge, Massachusetts, United States of America; 4 College of Fisheries and Ocean Sciences, Institute of Marine Science, Fairbanks, Alaska, United States of America; 5 Centre de recherche sur les biotechnologies marines, Rimouski, Québec, Canada; University of Maryland Center for Environmental Science, UNITED STATES

## Abstract

The toxic diatom genus *Pseudo-nitzschia* is distributed from equatorial to polar regions and is comprised of >57 species, some capable of producing the neurotoxin domoic acid (DA). In the Pacific Arctic Region spanning the Bering, Chukchi, and Beaufort seas, DA is recognized as an emerging human and ecosystem health threat, yet little is known about the composition and distribution of *Pseudo-nitzschia* species in these waters. This investigation characterized *Pseudo-nitzschia* assemblages in samples collected in 2018 during summer (August) and fall (October-November) surveys as part of the Distributed Biological Observatory and Arctic Observing Network, encompassing a broad geographic range (57.8° to 73.0°N, -138.9° to -169.9°W) and spanning temperature (-1.79 to 11.7°C) and salinity (22.9 to 32.9) gradients associated with distinct water masses. Species were identified using a genus-specific Automated Ribosomal Intergenic Spacer Analysis (ARISA). Seventeen amplicons were observed; seven corresponded to temperate, sub-polar, or polar *Pseudo-nitzschia* species based on parallel sequencing efforts (*P*. *arctica*, *P*. *delicatissima*, *P*. *granii*, *P*. *obtusa*, *P*. *pungens*, and two genotypes of *P*. *seriata*), and one represented *Fragilariopsis oceanica*. During summer, particulate DA (pDA; 4.0 to 130.0 ng L^-1^) was observed in the Bering Strait and Chukchi Sea where *P*. *obtusa* was prevalent. In fall, pDA (3.3 to 111.8 ng L^-1^) occurred along the Beaufort Sea shelf coincident with one *P*. *seriata* genotype, and south of the Bering Strait in association with the other *P*. *seriata* genotype. Taxa were correlated with latitude, longitude, temperature, salinity, pDA, and/or chlorophyll *a*, and each had a distinct distribution pattern. The observation of DA in association with different species, seasons, geographic regions, and water masses underscores the significant risk of Amnesic Shellfish Poisoning (ASP) and DA-poisoning in Alaska waters.

## Introduction

Harmful algal blooms (HABs) have been reported in the subpolar marine waters of southeast Alaska for decades, leading to negative impacts on human and/or wildlife health [1–3]. There is great concern that changing climate conditions could promote intensification and geographic expansion of HABs and their toxins, particularly in the Arctic waters north of the Bering Strait [4–9]. Efforts to monitor phytoplankton, including HAB species, in this area are a growing priority [4, 6, 9].

Diatoms are critical to biogeochemical cycling and food web dynamics in the Arctic and often dominate phytoplankton assemblages especially in productive, high nutrient waters [9–12]. Some species in the HAB-forming diatom genus *Pseudo-nitzschia* have been shown to produce domoic acid (DA), a neurotoxin that can cause Amnesic Shellfish Poisoning (ASP) in humans and Domoic Acid Poisoning (DAP) in wildlife [13, 14]. The first and only known cases of ASP occurred in eastern Canada in 1987 [13]. In contrast, DAP has been documented more widely in marine ecosystems, particularly in temperate waters along the Pacific coast of the United States [15]. Further north, the Alaska coastline spans both the North Pacific and Arctic oceans, bridging the biogeographic transition from temperate to polar “ecoregions” [16] that represent distinct marine temperature regimes [17]. A multi-year study found DA in 13 species of marine mammals from the Gulf of Alaska to the Beaufort Sea [3], and low DA levels have also been observed infrequently in other biota, including forage fish, invertebrates, and seabirds [6, 18]. Knowledge of the spatiotemporal distribution of *Pseudo-nitzschia* spp. in the Arctic and across these temperature regimes is limited, but critical for linking production and trophic transfer of DA.

The genus *Pseudo-nitzschia* consists of nearly 60 documented species, and occurs in estuarine, coastal, and oceanic environments [14, 19–22] as well as in association with sea ice [9]. Roughly half the known *Pseudo-nitzschia* species are toxic to varying degrees, and species composition is critical to understanding patterns of DA toxicity [14, 23–28]. Toxin production is highly variable across species and also within a species as it can be influenced by physiological status, growth conditions, and life cycle stage [14, 29–32]. Species often co-occur and have the potential to respond quickly to environmental changes in space and/or time [24, 25, 33]. Yet, species composition is often not routinely assessed since the genus contains cryptic and pseudo-cryptic species complexes that cannot be readily resolved without electron microscopy and/or molecular approaches [34–36].

Some early studies of *Pseudo-nitzschia* in Alaska waters utilized light microscopy to differentiate morphotypes based on cellular morphology [9, 37], however, more recent studies included additional taxonomic analyses to help resolve species. Thus far, several toxic species from temperate and/or subpolar Pacific regions (e.g., *P*. *delicatissima*, *P*. *granii*, *P*. *multiseries*, *P*. *pseudodelicatissima*, *P*. *pungens*, and *P*. *seriata*), as well as at least two species considered to be polar (*P*. *obtusa*, a toxic species previously described as a form of *P*. *seriata*; and *P*. *arctica*, a recently described non-toxic member of the *P*. *pseudodelicatissima* species complex) have been identified [9, 35, 37–40].

The Distributed Biological Observatory (DBO; https://arcticdata.io/catalog/portals/DBO) was established in 2010 to evaluate seasonal and interannual changes related to climate change—including rapidly warming temperatures and dramatic shifts and losses in seasonal sea ice extent—in the Pacific Arctic Region (PAR) [41, 42]. The PAR encompasses several interconnected subregions including the northern Bering Sea, the Chukchi Sea (including Point Barrow and Barrow Canyon), and the Beaufort Sea. The DBO includes transects visited by numerous programs to assess seasonal to interannual changes in primary productivity, biodiversity, and biomass “hotspots” and their underlying mechanisms [41–44]. Ocean currents, bathymetric features, and seasonal sea ice extent influence water mass characteristics, phytoplankton growth condition including nutrient status (i.e., oligotrophic to eutrophic) and light, and thus shape ecological niches for phytoplankton [11, 43–46]. Water masses of distinct origins (i.e., from the Atlantic, Pacific, and Arctic Oceans) occur in the PAR [47, 48]. Pacific water typically flows northward from the Bering Sea into the Chukchi Sea via the Bering Strait, with transport varying by season and year [48–50]. In recent years, the DBO has prioritized investigations of HABs in the PAR [4, 5].

*Pseudo-nitzschia* transport by ocean currents has been described in other regions [51–53] yet little is known about the spatiotemporal dynamics of *Pseudo-nitzschia* spp. and DA in the PAR. Species assemblages and toxicity have the potential to vary across water masses that have distinct geographic origins and that are advected into the region, but there may also be endemic and/or ice-dwelling species that contribute to localized DA production. As a first step towards understanding recent and future observations of DA in Alaska, the seasonal distribution and diversity of *Pseudo-nitzschia* species and DA across subregions and key water masses of the PAR were assessed in samples collected during summer (August) and fall (October-November) surveys in 2018. Sensitive analytical approaches were used to determine *Pseudo-nitzschia* species diversity (i.e., fingerprinting and sequencing environmental DNA, targeting the Internal Transcribed Spacer 1 region) and concentrations of particulate or pDA (measured using an ultra-high performance liquid chromatographic system coupled to a mass spectrometer). In addition, *Pseudo-nitzschia* abundance was assessed using an Imaging FlowCytobot (IFCB) that continuously sampled seawater via a flow-through system during the summer survey. Together, these observations provided unprecedented baseline information about *Pseudo-nitzschia* species composition and potential toxicity in this dynamic and rapidly changing region.

## Materials and methods

### Study site description

**Fig 1:**
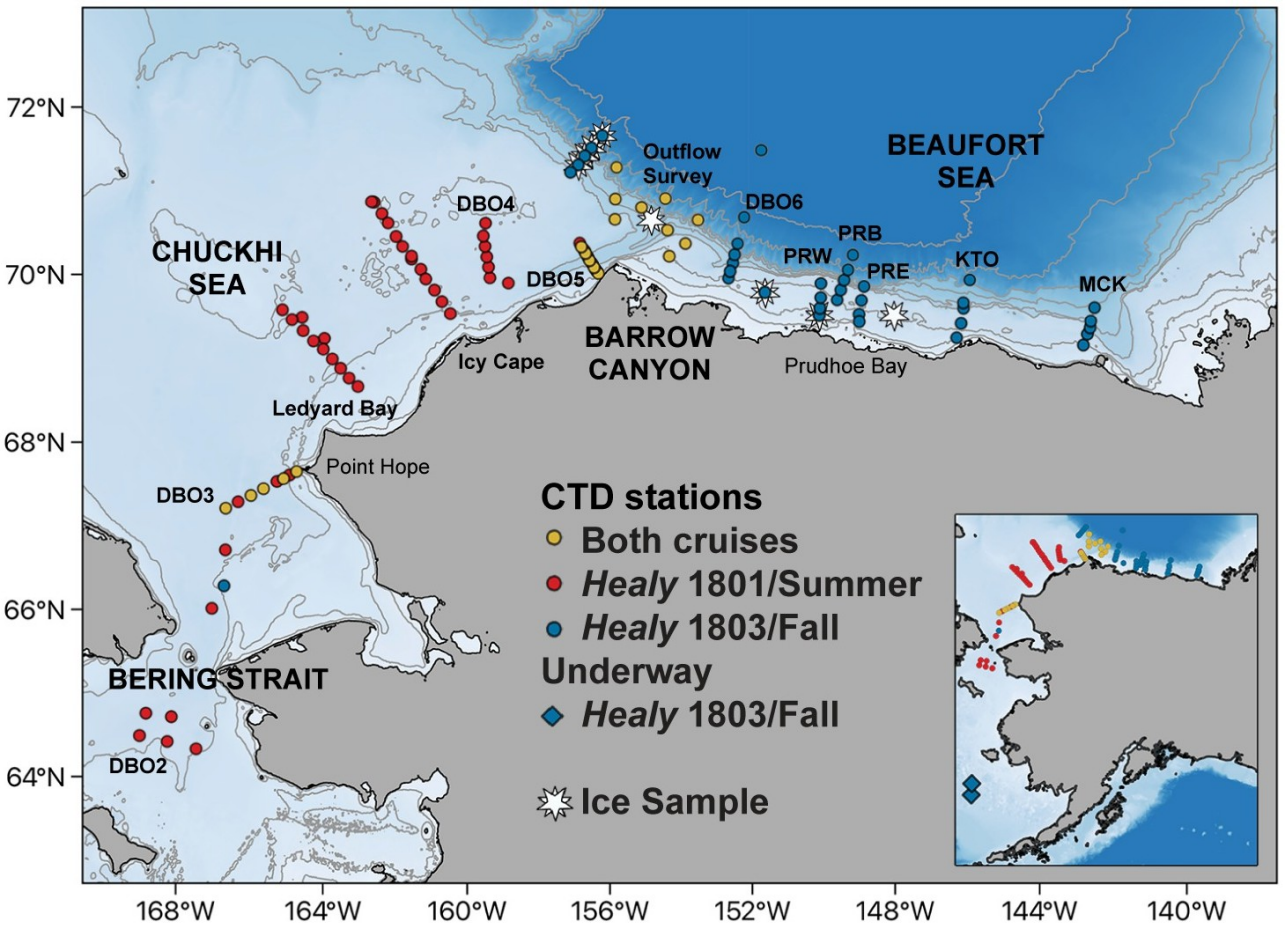
Station map. Locations of stations sampled during summer (*Healy* 1801; red circles), fall (*Healy* 1803; blue circles), or both cruises (yellow circles). Underway stations sampled (blue diamonds) and sea ice samples (white stars) were collected on the fall *Healy* 1803 cruise. Major circulation pathways are highlighted separately [55].

Surface and subsurface samples (n = 297) were collected aboard the United States Coast Guard Cutter (USCGC) *Healy* in 2018 during summer (cruise 1801, August 4–24) and fall (cruise 1803, October 24 –November 18) surveys (Fig 1, Table 1, and S1 Table). With the exception of figures and tables, all subsequent mentions of either survey will refer to summer or fall only. Spatial coverage (Fig 1) was mapped in QGIS (v. 3.16.11), an open-access software, with the bathymetric grid from the General Bathymetric Chart of the Oceans (GEBCO) [54]. Raster-to-vector conversion was used to pull bathymetric contours and shapefiles for the base map, overlaid with station locations. The summer cruise was part of the DBO program, with 170 samples collected from 63 sites, including the northern Bering Sea transition into the Bering Strait (DBO2); the Chukchi Sea at mooring sites and along five transect lines: DBO3, Ledyard Bay (LB), Icy Cape (IC), DBO4; and the vicinity of Barrow Canyon: DBO5 and Outflow Survey (OS). The fall cruise, associated with the Arctic Observing Network (AON), involved the collection of 127 samples from 56 sites, including the Chukchi Sea (DBO3), the vicinity of Barrow Canyon (DBO5 and OS), and the Beaufort shelf: DBO6, Prudhoe Bay East (PBE), Prudhoe Bay (PRB), Prudhoe Bay West (PBW), Kaktovik (KTK), and Mackenzie Canyon (MCK). Sea ice cover was obtained from the National Snow and Ice Data Center (NSIDC), and varied significantly between the two surveys and was dynamic, with newly forming ice observed during the fall survey (S1 Fig). Ice was sampled at a small subset of sites from just west of Point Barrow into the Beaufort Sea. Overall, five geographic sub-regions were sampled: Bering Sea, Bering Strait, Chukchi Sea, Point Barrow/ Barrow Canyon (referred to as Barrow Canyon), and Beaufort Sea. Only sites from north of the Bering Strait (DBO3) and the Barrow Canyon sub-regions (DBO5 and OS) were sampled during both surveys. S1 Table includes a detailed description of all stations and parameters sampled during both cruises.

**Table 1:** Summary of sampling locations. The number of stations sampled during summer *Healy* 1801 and fall *Healy* 1803 cruises are grouped by subregion. Common transects between both cruises are in bold. See S1 Table and text for detail.

Subregion	Transect	*Healy* 1801- summer	*Healy* 1803 –fall
		# stations (# samples)	# stations (# samples)
Bering Sea	Transit/Flow		2 underway samples
Bering Strait	DBO2	4 (10)	-
Chukchi Sea	Transit	2 (7)	
	Test Cast	-	1 (3)
	**DBO3**	**8 (24)**	**5 (14)**
	Ledyard Bay (LB)	8 (27)	-
	Icy Cape (IC)	11 (30)	-
	DBO4	6 (16)	-
	Moorings (C)	5 (15)	-
Barrow Canyon	**DBO5**	**10 (22)**	**5 (10)**
	**Outflow Survey**	**9 (19)**	**14 (35)**
Beaufort Sea	DBO6	-	6 (12)
	BW1	-	1 (3)
	Fill-7		1 (1)
	Prudhoe Bay East (PRE)	-	4 (8)
	Prudhoe Bay (PRB)	-	5 (9)
	Prudhoe Bay West (PRW)	-	4 (9)
	XBT	-	1 (1)
	Kaktovik (KTO)	-	5 (10)
	Mackenzie Canyon (MCK)	-	5 (10)

### Vertical profiling and water mass designations

At each station, vertical profiling was conducted using a Sea-Bird Electronics 911+ conductivity-temperature-depth (CTD) instrument. Data from downcast profiles were processed using Sea-Bird’s software as described by Pickart et al. (2019) [48]. Parameters measured directly or calculated from sensor data included potential temperature referenced to the sea surface (T), practical salinity (S), potential density referenced to the sea surface (referred to as density), chlorophyll *a* fluorescence, and transmissivity. Measurements of T and S during the field surveys were used to assign each sample from 1801 and 1803 to one of five water masses: Alaska Coastal Water (ACW), Bering Summer Water (BSW), Melt Water / River Water (MWR), Newly Ventilated Winter Water (NVWW), and Remnant Winter Water (RWW); see S2 Fig. The T/S boundaries defining these water masses have been used in many previous studies [50, 56–59]. Plots showing T/S at each station sampled were generated using MATLAB (2021b).

### Discrete sampling and nutrient/chlorophyll analyses

A rosette of Niskin bottles was used to collect discrete surface and subsurface seawater samples for nutrient, chlorophyll *a*, genetic, toxin, and microscopy analyses. During the fall cruise, additional samples were collected while underway via the flow-through seawater system on the *USCGC Healy*, and were partitioned for genetic, microscopy, and particulate DA (pDA) analysis only. Also during fall, several sea-ice samples were opportunistically collected via a net while the ship was stationary. Ice samples were maintained in the dark at room temperature until melted, and then similarly partitioned.

For size-fractionated chlorophyll *a* analysis, seawater samples (0.5 L) from the summer survey only were filtered sequentially onto 5 μm and then 0.7 μm pore-size glass fiber filters and frozen at -20°C until analysis. Chlorophyll *a* was extracted with acetone (90%), measured on a Turner Designs 10 AU fluorometer, and samples were then acidified to assess phaeopigment concentrations following Parsons et al. (1984) [60]. Total extracted chlorophyll *a* and phaeopigment concentrations were then calculated by adding the values from each size fraction. For determination of the chlorophyll maximum, chlorophyll fluorescence from CTD profiles was used.

Seawater samples were collected for genetic analysis at each site, generally targeting the surface and 10 m depths; for a subset of sites, especially during summer, additional subsurface samples including the chlorophyll maximum (chl max) were collected. Sea ice and underway samples were collected during fall. Samples (350 mL to 500 mL) were filtered onto 0.45 μm pore size nitrocellulose filters (Millipore) and frozen shipboard at −80°C, shipped on dry ice, and maintained in the laboratory at −80°C until extraction. For microscopy-based analyses, a 125 mL seawater or melted sea-ice sample was preserved in Lugol’s iodine solution (1.6% final concentration).

For particulate DA analysis, seawater and melted sea-ice samples (350 mL to 500 mL) were collected in tandem with genetic samples and filtered onto Whatman^®^ GF/F filters, placed into cryovials, frozen and shipped on dry ice, and stored at -80°C until extraction.

### Automated Ribosomal Intergenic Spacer Analysis (ARISA)

Genomic DNA was extracted from filters using a DNeasy Plant Mini Kit (Qiagen Inc.) according to provided instructions, eluted in a final volume of 150 μL AE buffer, and quantified using Picogreen (Invitrogen) with a Synergy H1 plate reader (BioTek Instruments, Inc.). Amplification for ARISA was conducted following published protocols that used 10 ng per PCR with each sample run in triplicate [24], however, most samples did not successfully amplify. Instead, ARISA was conducted using a two-step nested PCR. The first PCR utilized oligonucleotides 18SF-euk (5′-CTTATCATTTAGAGGAAGGTGAAGTCG-3′) and 5.8SR-euk (5′-CTGCGTTCTTCATCGTTGTGG-3′) to amplify the full ITS1 region [23]. Genomic DNA (1 μL equaling <1–11 ng) was added to triplicate 20 μL PCRs that consisted of 2.5 mM deoxynucleoside triphosphates, 0.4 μM of each primer, 0.75 U of Apex Taq polymerase, 2 mM of MgCl_2_, and 1 x standard reaction buffer (Apex Bioresearch Products). Amplifications consisted of a 2–minute denaturation step at 94°C, then 32 cycles of 30 seconds at 95°C, 30 seconds at 67°C, and 60 seconds at 72°C, followed by a final 10–minute extension at 72°C. From this first PCR, products were quantified using PicoGreen and 1 μL was added to a second set of 20 μL PCRs utilizing previously designed genus-specific oligonucleotides PnallF (5′-TCTTCATTGTGAATCTGA-3′) and FAM-labeled Pnall R (5′-CTTTAGGTCATTTGGTT-3′; Eurofins Genomics) [24]; other reagents were added at similar concentrations to those described above and were cycled similarly to the first PCR, with the exception of the annealing temperature (50.6°C). Sterile molecular grade water was used for blanks associated with one-step and both steps of the nested PCRs. Resulting PCR products were purified using MultiScreen PCRμ96 filter plates (Millipore). During purification, triplicate PCR products for each sample were pooled and again quantified with PicoGreen. After standardizing PCR products to 1 ng μL^-1^, 1 μL of each sample was analyzed on an Applied Biosystems 3730 XL DNA Analyzer (University of Illinois DNA Core Sequencing Facility) using a LIZ600 size standard for DNA fragment analysis. To distinguish putative amplicon sizes, DAx software (Van Mierlo Software Consultancy) was used to analyze electropherograms. Proportions of taxa within each sample were assessed by calculating each individual taxon peak height divided by each sample’s total peak height; only peaks that comprised ≥3% of the total peak height fluorescence for that sample were further analyzed. In order to accurately match ARISA amplicon sizes to ITS1 sequence lengths, 4 bp were manually added to each amplicon size associated with a peak, based on comparisons to reference sequences (including those generated herein).

Prior studies highlight the efficacy of ARISA in reflecting proportions of ITS1 copies added to the PCR rather than absolute cellular abundance, in part because cellular and genome sizes vary across species [24]; for semi-quantitative comparison of species assemblages across samples, patterns described herein thus refer to dominance of the ARISA signal rather than numerical dominance. Bar charts of species data were generated using Sigmaplot and labels were added in Adobe Illustrator. Maps of species distribution were generated using a gridding resolution of 0.2° in MATLAB (2021b), with bathymetry data obtained from the built-in data package, ETOPO2 (https://sos.noaa.gov/catalog/datasets/etopo2-topography-and-bathymetry-natural-colors/).

### Sequencing

To generate material for sequencing, genomic DNA (1 μL equaling <1–11 ng) was amplified using PCR with the 18SF-euk and 5.8SR-euk oligonucleotides as described above; resulting PCR products were run on a 3.5% agarose gel. Fragments were individually picked from the gel using sterile micropipette tips. Each gel-pick was melted in 10 μL of sterile molecular grade water for five minutes at 40°C and 1 μL of the melted gel/sterile water mixture was amplified by PCR using the PnAll F/R oligonucleotides, and added to a 3.5% agarose gel to verify the presence of a single band and estimate product size. The remaining PCR products were purified using ExoSap-It Express PCR Product Cleanup Reaction (Applied Biosystems, Waltham, MA) according to manufacturer provided instructions. Purified product was sequenced on a 3730 XL DNA Analyzer (Applied Biosystems) by Eurofins Genomics LLC (Louisville, KY). Sequences were subsequently checked for quality and analyzed using Sequencher (Gene Codes Corporation, Ann Arbor, MI), and identified using BLASTn [61] to query the National Center for Biotechnology Information’s (NCBI) GenBank nucleotide database. Newly generated sequences were trimmed to remove primer regions and submitted to GenBank (accession numbers OK474255-OK474278).

### Microscopy analyses

A subset of 23 Lugol’s-preserved surface and chlorophyll maxima samples from summer and fall surveys were settled (3–100 mL) for examination and enumeration via inverted light microscopy, and to identify candidate samples for analysis with electron microscopy. Cells were not readily observed in these samples, thus further analysis was not conducted.

During the summer cruise (1801), an Imaging FlowCytobot (IFCB) was configured to sample continuously every ~25 minutes from the shipboard underway seawater system (~2 m depth). *Pseudo-nitzschia* spp. cells were identified and chain lengths counted through manual annotation of the image time series. IFCB sample bin locations were assigned through comparison of time stamps to ship positions recorded by USCGC *Healy*. Cell lengths and widths were estimated using “v3” blob and feature extraction available from https://github.com/hsosik/ifcb-analysis.git and converted to real dimensions using an estimate of 2.77 pixels/micron. Plots summarizing IFCB data collection and results were generated using MATLAB (2021b) and merged with cell micrographs using Adobe Photoshop.

### Toxin analysis

Prior to extraction, filters for DA analysis were placed in individual 15-mL polypropylene centrifuge tubes. Domoic acid was extracted by adding 5 mL of 20% aqueous MeOH (ACS grade; ThermoFisher Scientific, Waltham, MA, USA); 4 mL was added directly to centrifuge tubes containing filters and 1 mL was used to rinse each cryovial and forceps before adding to these tubes. Then, samples were vortexed for 2 min, and centrifuged at 4 °C and 3500 x g for 15 min. The supernatant was then transferred to a glass vial and stored in the dark at -20°C until analysis.

Extracts were filtered through 0.22 μm PVDF syringe filters and analyzed using an Acquity I-Class ultra-high performance liquid chromatographic (UPLC) system coupled to a Xevo TQ-XS tandem triple quadrupole mass spectrometer (MS) equipped with a UniSpray^®^ ion source (Waters, Milford, MA, USA). The UPLC was fitted with an Acquity UPLC BEH C18 1.7 μm column (2.1 x 100 mm). The injection volume was 5 μl, with a flow rate of 0.8 ml/min and a column oven temperature of 40°C. Mobile phase A consisted of a 0.1% formic acid solution in water (LC-MS grade), and mobile phase B was acetonitrile with 0.1% formic acid. Acetonitrile, formic acid, and water used for analysis were purchased from Thermo Fisher Scientific. The initial gradient condition was 5% mobile phase B, followed by a linear gradient to 95% B at 2 min, which was held for 0.5 min. Then, a linear gradient was used to 30% B at 4 min, which was held for 1.5 min before returning to initial conditions at 6 min and reconditioning the column for 1 min. The MS parameters were: impactor voltage 1kV, cone voltage 28 V, desolvation temperature 600°C, cone gas flow 150 L/h, and desolvation gas flow 900 L/h. MRM transitions from the protonated DA ion were monitored for the following transitions: *m/z* 312 > 193, *m/z* 312 > 248, and *m/z* 312 > 266; and collision energies were optimized for each precursor/product pair. A certified reference standard solution of DA (National Research Council, Halifax, Canada) was used to generate a standard curve for quantitation of DA. Samples with trace levels of DA had concentrations below the limit of quantitation based on this standard curve.

### Statistical analyses

Species proportions derived from ARISA and environmental measurements generally did not exhibit normal distributions based on a preliminary examination, thus nonparametric tests were used for statistical comparisons. Spearman’s Rank Correlation Coefficient was used to evaluate relationships among environmental variables (temperature, salinity, pressure, dissolved oxygen, in situ fluorescence, concentrations of extracted chlorophyll *a* and phaeophytin, transmissivity, and domoic acid), among taxa identified with ARISA, as well as between taxa and environmental variables. Spearman’s Rho was calculated using IBM SPSS Statistics for Windows (IBM Version 26.0, IBM Corp., Armonk, NY, USA). To explore space-time correlation trends in species space, and association with their underlying environmental variables, the following statistical analyses were performed with Primerv7 software with Permanova+ [62]: ordination with multi-dimensional scaling using Principal Coordinates Analysis (PCO) and permutation-based hypothesis testing (of differences between and among groups of samples from summer and fall and/or distinct sub-regions) using Analysis of Similarities (ANOSIM). The PCO and ANOSIM analyses were carried out for all discretely sampled locations (1801+1803: all taxa, 286 samples) and for each survey separately (1801: summer taxa, 170 samples; 1803: fall taxa, 116 samples), with the exception of those 11 ice and flow-through samples that were excluded from these analyses. For the PCO, the Bray Curtis similarity index was utilized for species data. Environmental variables used for both analyses (temperature, salinity, and chlorophyll *a* fluorescence) were selected to avoid limitations due to missing data (pigments and domoic acid were excluded for this reason) or redundancy demonstrated by those parameters found to be highly correlated in the Spearman’s analysis.

## Results

### Physical environment and water masses

During the summer cruise, sea ice was not observed, consistent with a decrease in extent from August to September (S1 Fig). In contrast during fall, air temperatures were consistently below freezing and sea ice formation observed during sampling allowed collection of a small number of ice samples (S1 Fig, S1 Table). Considering all sampling locations, ranges of latitude and longitude nearly doubled between the summer (69.67 to 72.62°N and -169.93 to -153.36°W) and fall (57.79–73.01°N and -169.0 to -138.93°W) cruises. Both surveys encompassed substantial gradients in T and S (Table 2); summer samples (Bering Strait to Barrow Canyon) encompassed a 13.32°C (T: -1.62°C to 11.70°C) temperature range compared to 7.64°C (T: -1.79°C to 5.85°C) in fall (Chukchi Sea to the Beaufort Shelf). The opposite was true for salinity which spanned a narrower range in summer (26.8 to 32.9) compared to fall (22.9–32.6).

**Table 2:** Water mass characteristics during summer (*Healy* 1801) and fall (*Healy* 1803). Number of samples per cruise (in parentheses after water mass names), and minima (min) and maxima (max) for temperature, salinity, dissolved oxygen, and chlorophyll *a* are included.

**Summer *Healy* 1801**	**ACW (84)**		**BSW (24)**		**MWR (44)**		**NVWW (1)**		**RWW (17)**	
	min	max	min	max	min	max			min	max
Temperature (°C)	3.81	11.70	1.08	8.57	-0.89	11.02	-1.62		-1.46	-0.23
Salinity (psu)	30.07	31.92	31.28	32.86	26.83	29.86	32.42		31.82	32.57
Oxygen (mg L^-1^)	6.13	7.73	5.99	8.94	6.43	9.44	6.70		6.38	11.20
Fluorescence	1.41	9.84	1.99	40.28	2.06	5.22	3.87		3.23	28.49
Chlorophyll (mg m^-3^)	0.21	2.40	0.20	15.19	0.18	1.30	0.99		0.26	6.96
Subregions	BE, C, BA		BE, C		C, BA		C		C, BA	
**Fall *Healy* 1803**	**ACW (3)**		**BSW (16)**		**MWR (73)**		**NVWW (20)**		**RWW (4)**	
	min	max	min	max	min	max	min	max	min	max
Temperature (°C)	5.51	5.85	0.87	4.13	-1.69	-0.01	-1.79	-1.61	-1.59	-1.07
Salinity (psu)	30.68	31.67	30.02	32.57	22.88	31.48	31.58	32.33	31.57	32.20
Oxygen (mg L^-1^)	5.50	6.86	5.85	8.11	7.96	9.02	7.55	8.52	7.38	8.14
Fluorescence	2.91	6.13	2.11	3.15	1.85	3.10	2.10	2.72	2.24	2.45
Chlorophyll (mg m^-3^)	N/A*	N/A	N/A	N/A	N/A	N/A	N/A	N/A	N/A	N/A
Subregions	C		C, BA		BA, BF		BA, BF		BA, BF	

Water Masses: ACW, Alaskan Coastal Water; BSW, Bering Summer Water; MWR, Melt Water / River Water; NVWW, Newly Ventilated Winter Water; RWW, Remnant Winter Water. Sub-regions: BE, Bering Strait; C, Chukchi Sea; BA, Barrow Canyon, BF, Beaufort Sea.

*N/A indicates not available due to lack of sampling.

Measurements of T and S were used to associate each seawater sample with one of the water masses defined above (see S2 Fig, Table 2), except for flow through samples. Overall, samples corresponding to ACW, BSW, and RWW were more prevalent in summer, whereas MWR and NVWW were more prevalent in fall (Table 2). The majority of the 286 samples (41%) were associated with Melt Water/River Water (MWR), which also displayed the broadest T and S ranges (S2 Fig). Samples designated as MWR originated from all areas sampled except for the Bering Sea. Observations of MWR were more prevalent in samples collected in the upper water column (surface and 10m). Alaskan Coastal Water (ACW) included the warmest samples analyzed from each survey and was associated with 30% of samples from all sub-regions except the Beaufort Sea. During summer, ACW was particularly prevalent at the surface in the Bering Strait, across depths in the Chukchi Sea, and at depth in the Barrow Canyon. During fall, this water mass was only associated with the most southern Chukchi Sea sampling site (at all depths). Bering Summer Water (BSW) represented 14% of samples, including those from the Bering Strait, Chukchi Sea (during summer only), and Beaufort Sea (during fall only). This water mass was generally more prevalent at depth than the surface during summer. The coldest and saltiest water masses, Remnant Winter Water (RWW) and Newly Ventilated Winter Water (NVWW), were not observed in the Bering Sea or Bering Strait, and each represented 7% of samples, including summer Chukchi Sea samples, summer and fall Barrow Canyon samples, and fall Beaufort Sea samples. All summer observations of RWW or NVWW occurred at 20m or deeper; in fall, NVWW or RWW sites were close to shore, with RWW still only observed in subsurface samples.

Chlorophyll *a* fluorescence values, measured with the CTD, displayed a broader range during summer (1.41–40.28 mg m^-3^) than fall (1.85–6.13 mg m^-3^); discrete chlorophyll concentrations were measured in summer only, with maxima observed in BSW (>15.0 mg m^-3^; Table 2). Dissolved oxygen concentrations (between 5.50 and 11.20 mg L^-1^) were fairly comparable between cruises, with the highest and lowest concentrations respectively associated with RWW measured during summer and ACW measured during fall (Table 2).

### Genetic characterization of taxa

The ARISA method coupled with sequencing was used to characterize *Pseudo-nitzschia* assemblages. Eight of the 17 ARISA peaks observed in electropherograms (Table 3) were identified using direct sequencing of PCR products: *P*. *delicatissima* (168 base pairs [bp]), *P*. *obtusa* (147 bp), *P*. *pungens* (142 bp), *P*. *granii* (180 bp), *P*. *arctica* (178 bp), *P*. *seriata* “type 1” (151 bp), *P*. *seriata* “type 2” (150 bp), and one species from a closely related genus, *Fragilariopsis oceanica* (190 bp). *In silico* analysis indicated that *F*. *oceanica* was the only *Fragilariopsis* species that was a perfect match with the ARISA primers, however, a few species from other polar or sub-polar genera predicted to have 2 or fewer mismatches to the Pnall primers shared ARISA sizes with the unidentified peaks (S2 Table). Two species, *P*. *granii* and *P*. *arctica*, had ARISA sizes that were ~1 bp greater than expected from sequences but also had a 1 bp mismatch to the reverse Pnall primer that likely caused this offset. *P*. *seriata* “type 1,” previously recorded in the Atlantic, had one additional nucleotide relative to *P*. *seriata* “type 2” leading to a 1 bp size difference (151 and 150 bp, respectively) that was verified by both ARISA and sequencing. In the Pacific Northwest, both *P*. *australis* and *P*. *seriata* “type 2” share a 150 bp ARISA fragment size, and although *P*. *australis* was not picked up via sequencing, the possibility that it was also present in samples with a 150 bp peak cannot be excluded based on the analyses conducted. Nine additional amplicons were characterized by ARISA only and could represent additional *Pseudo-nitzschia* species or populations (160 bp, 161 bp, 165 bp, 170 bp, 172 bp, 196 bp, 208 bp, 235 bp, and 240 bp). Based on prior observations in NE Pacific samples [24, 63], it is possible that the 196 bp peak could represent *P*. *heimii* (195 bp), *P*. *americana* (196 bp), or another taxon, but without further confirmation, peak size only is referenced here.

**Table 3:** Summary of species and sequences generated and used for identification of ARISA sizes in summer (*Healy* 1801) and fall (*Healy* 1803) samples.

Taxon	Domoic acid producer	Predicted/ observed ARISA length (bp)	# seqs (this study)	Genbank Accession number (this study)	Cruise	Station	Depth	Most similar Genbank sequence	Percent identity (# bp)	Geographic origin of similar Genbank sequence
*P*. *pungens*	Yes	142/142	3	OK474255	1801	LB-7	S	MK106646.1	100% (107 bp)	Adriatic Sea, Slovenian coast [64]
				OK474256	1801	LB-6	S			
				OK474257	1803	DBO3-3	10m			
*P*. *obtusa*	Yes	147/147	3	OK474258	1801	DBO4-4	25.5m/ CM	DQ062667.1	100% (112 bp)	Tromsø, Norway, Atlantic Arctic [38]
				OK474259	1801	DBO4-3	15.6m/ CM			
				OK474260	1801	DBO4-5	25.4m/CM			
*P*. *seriata* “type 1”	Yes	151/151	2	OK474261	1803	PRB-12	10m	JF974030.1	100% (116 bp)	Nuuk, Greenland, Atlantic Arctic [65]
				OK474262	1803	MCK-3	S			
*P*. *seriata* “type 2”	Yes	150/150	2	OK474264	1803	T-3	Flow	DQ996022.1	100% (115 bp)	Washington, USA [23]
				OK474263	1803	Test Cast	10m			
*P*. *delica-tissima*	Yes	168/168	7	OK474265	1801	C-4	S	KR053128.1	100% (133 bp)	Washington, USA [66]
				OK474266	1801	OS4-7	10m			
				OK474267	1801	LB-7	S			
				OK474268	1801	OS4-2	20.5m/ CM			
				OK474269	1801	DBO5-9	S			
				OK474270	1803	DBO6-4	10m			
				OK474271	1803	T-3	Flow			
*P*. *arctica*	No	177/178	2	OK474272	1801	DBO4-6	S	KT589421.1	100% (142 bp)	Disko Bay, West Greenland [35]
				OK474273	1803	PRB-12	10m			
*P*. *granii*	Yes	179/180	1	OK474274	1803	DBO6-4	10m	KT948063.1	100% (144 bp)	Beaufort Sea [40]
*Fragilariopsis oceanica*	No	190	4	OK474275	1801	DBO4-1	S	GU170661.1	99% (154/ 155 bp)	Canadian Arctic, Beaufort Sea [67]
				OK474276	1801	LB-11	30m		100% (155 bp)	
				OK474277	1803	PRW-1	Ice			
				OK474278	1803	BW1-1	10m			

bp, base pairs; seqs, sequences; S, surface; CM, chlorophyll *a* fluorescence maximum

The majority of samples (n = 250) contained three or fewer peaks. A few fall samples reached the maxima of six peaks, each representing a distinct water mass and location (BSW/DB03, RWW/PRW10, and Transit 3). Of the 17 fragments, seven were common to both cruises: *P*. *delicatissima* (present in 256 samples), *P*. *obtusa* (in 76 samples), *P*. *pungens* (in 55 samples), *P*. *granii* (in 108 samples), *P*. *arctica* (in 70 samples), *Fragilariopsis oceanica* (in 97 samples), and the 170 bp amplicon (in 6 samples). Several unidentified amplicons (160 bp, 161 bp, 165 bp, 172 bp, 235 bp, and 240 bp) occurred exclusively in summer samples, and four taxa occurred only in fall samples (196 bp, *P*. *seriata* “type 2,” *P*. *seriata* “type 1,” and 208 bp). With the exception of the 161 bp fragment, which occurred in 18 samples, other unidentified fragments were observed in only 2–7 samples, and typically were not dominant, making it difficult to further confirm peak identity via the sequencing approach used herein. Seventeen and fifteen samples respectively were populated with *P*. *seriata* "type 1" or *P*. *seriata* “type 2.” Interestingly, 150 bp and 151 bp peaks (and their corresponding sequences) did not coincide in any samples, subregions, or water masses.

Six taxa were observed in the nine opportunistically collected sea ice samples from the fall survey. *Fragilariopsis oceanica* was the only species found in all ice samples, but most had 2–3 *Pseudo-nitzschia* species as well, including *P*. *arctica*, *P*. *delicatissima*, *P*. *granii*, *P*. *obtusa*, and/or *P*. *seriata* “type 1.” Seven of the nine samples had complementary surface samples and ice samples generally harbored more taxa than water samples. For example, five samples along transect OS5 had paired samples, and *F*. *oceanica* was absent from all water samples but present in ice samples, and *P*. *arctica* was present in three ice samples but not the complementary water samples.

### Taxa distribution patterns

**Fig 2:**
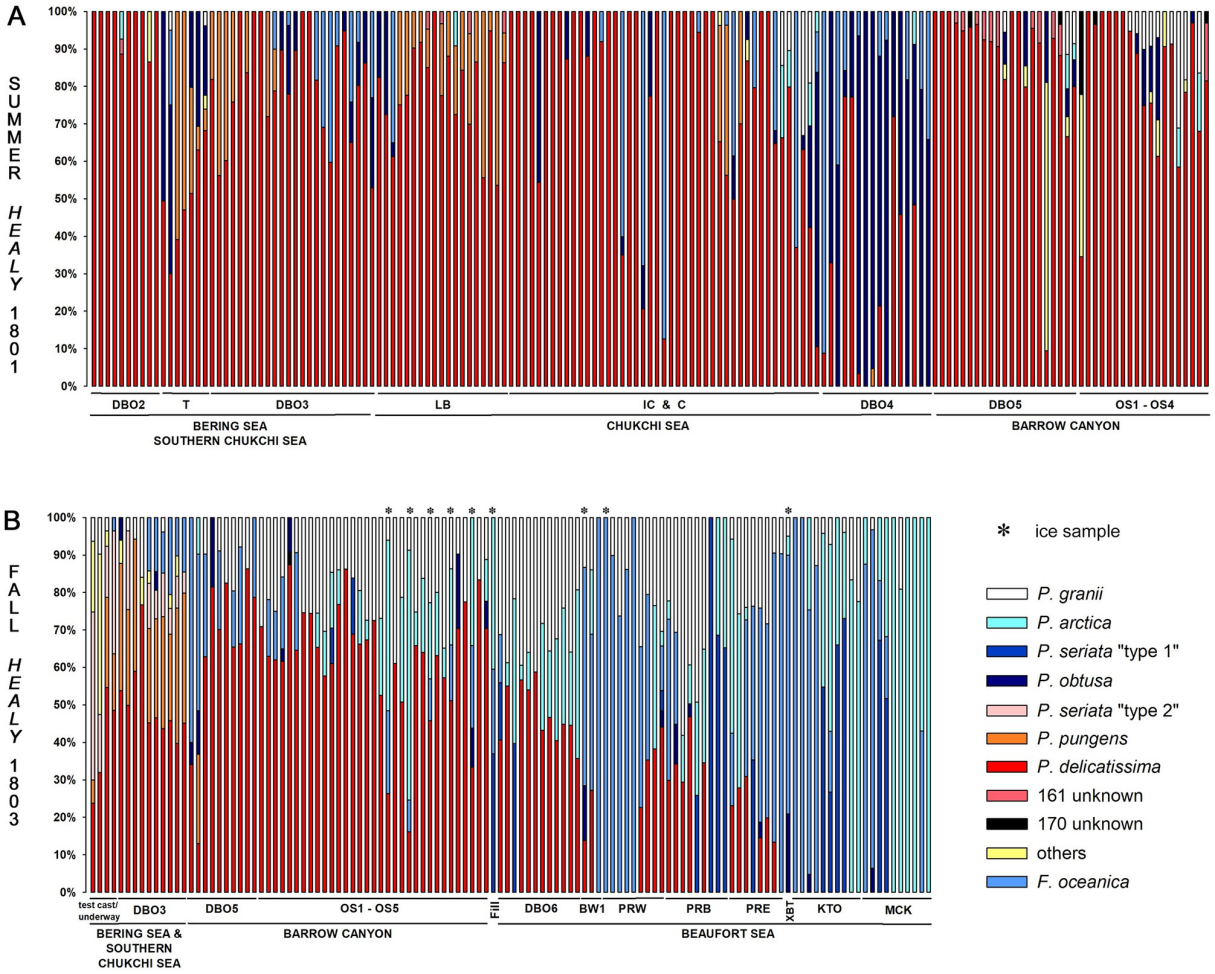
Bar plots of relative taxa abundance based on ARISA. Taxa are shown for stations sampled during: A) the summer cruise (surface, 10m, chlorophyll maximum) and B) the fall cruise (surface, 10m, and ice samples; the latter are denoted by an ice crystal). Category “others” reflects the sum of taxa with less than 3% frequency of occurrence across samples. Acronyms on x-axis refer to transects covered during field collections (see Table 1) with corresponding subregions indicated.

**Fig 3:**
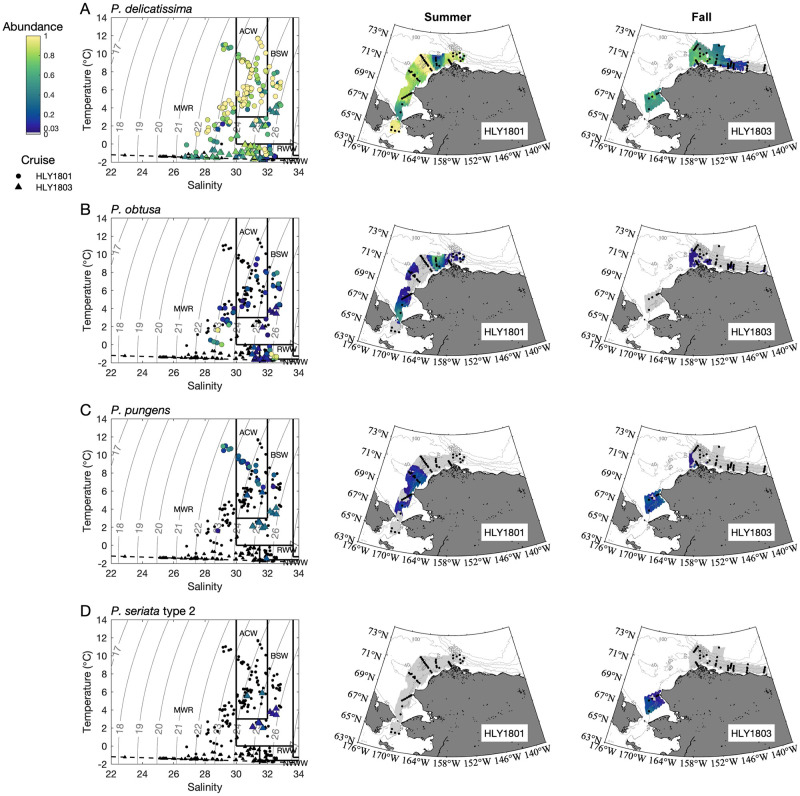
Distribution of species generally more prevalent in warmer water masses. Relative abundance of A) *P*. *delicatissima*, B) *P*. *obtusa*, C) *P*. *pungens*, and D) *P*. *seriata* “type 2” plotted in temperature and salinity space for all collection depths (left panels; solid lines show water mass boundaries, dotted lines show potential density, and dashed lines show freezing point of seawater) and plotted as contour plots for 10 m depth for summer (*Healy* 1801; middle panels) and fall (*Healy* 1803; right panels). Stations sampled during the respective cruises are indicated by black circles on the maps. Water mass abbreviations are the same as in Table 2.

**Fig 4:**
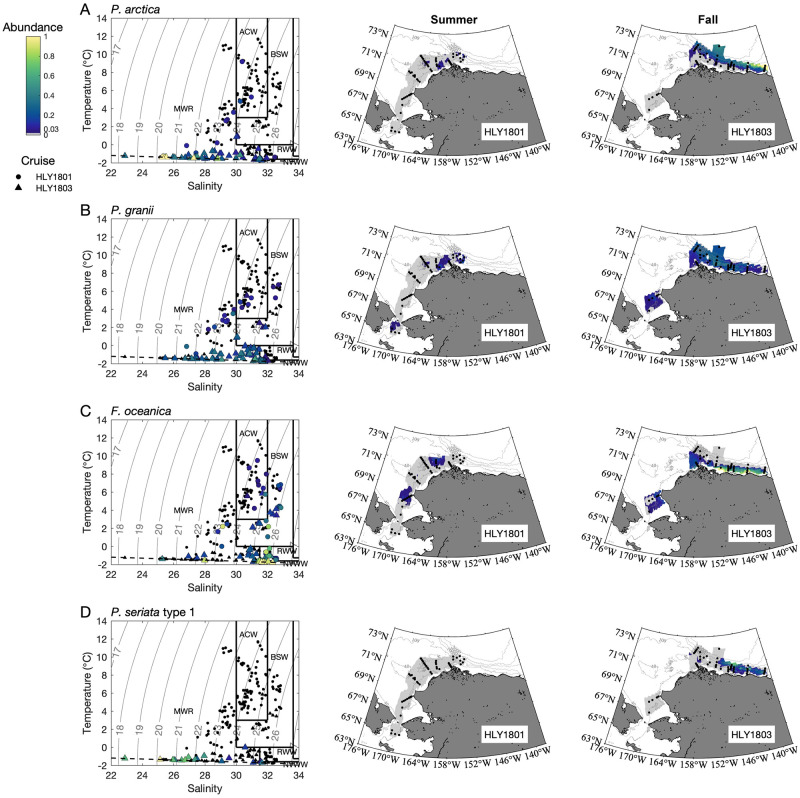
Distribution of species generally more prevalent in cooler water masses. Relative abundance of A) *P*. *arctica*, B) *P*. *granii*, C) *F*. *oceanica* and D) *P*. *seriata* “type 1” plotted in temperature and salinity space for all collection depths (left panels; solid lines show water mass boundaries, dotted lines show potential density, and dashed lines show freezing point of seawater) and plotted as contour plots for 10 m depth for summer (*Healy* 1801; middle panels) and fall (*Healy* 1803; right panels). Stations sampled during the respective cruises are indicated by black circles on the maps. Water mass abbreviations are the same as in Table 2.

**Fig 5:**
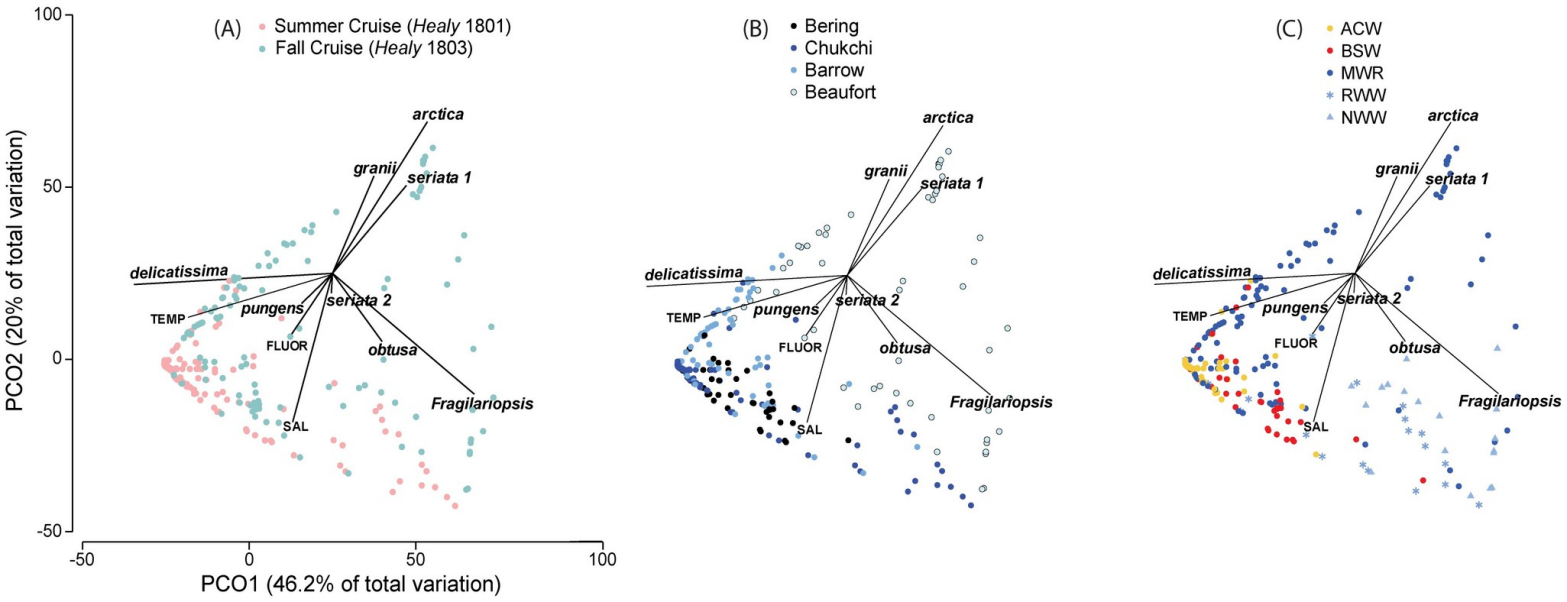
Biplots resulting from the PCO analysis. For both cruises (summer *Healy* 1801 and fall *Healy* 1803) combined (representing 17 taxa, 286 samples), the projection of samples (symbols) according to (a) cruise, (b) subregion, and (c) water masses is shown. Analysis performed in the taxa space (vectors) with environmental variables (vectors) independently calculated and overlaid on the biplot (TEMP = temperature, FLUOR = fluorescence, SAL = salinity). Only taxa for which identification has been validated by sequencing were included, *Pseudo-nitzschia* showing only the specific epithet for sake of simplicity.

Patterns of taxa occurrence were examined using spatial and statistical approaches. In general, assemblages detected by ARISA varied temporally (Fig 2), spatially (Figs 2–4), and across water masses (Figs 3–5; Tables 4 and 5; S3–S5 Tables). Spearman’s analysis revealed trends across the most commonly observed taxa (S4 Table) and/or environmental parameters (Table 4, S5 Table), and helped evaluate the subset of parameters and species to include in the PCO analysis. ANOSIM helped evaluate the magnitude of spatiotemporal and water mass (S6 Table) contributions to assemblage differences, and PCO analysis synthesized environmental structure underlying distribution patterns (Fig 5). Distribution patterns were also summarized with respect to each taxon relative to temporal, spatial, and water mass occurrence (Table 5).

**Table 4:** Summary of Spearman’s correlation coefficients (rho) between various environmental factors and selected diatom species from both surveys.

Taxa observed	Num	Lat*	Long	Pres	Temp	Sal	Chl *a*	Phaeo	Trans	Fluor	Oxy	DA
*P*. *delicatissima*	256		-0.448		**0.647**				**0.242**	**0.254**	-0.497	-0.273
*P*. *granii*	108	**0.38**	**0.513**	**-0.256**	-0.564	-0.202	-0.173	-0.217	**0.308**	-0.5	**0.481**	-0.134
*F*. *oceanica*	97	-0.128	**0.172**	**0.117**	-0.421	**0.465**	**0.172**	**0.216**	-0.373	-0.14	**0.132**	
*P*. *obtusa*	76		-0.138	**0.251**		**0.283**						**0.427**
*P*. *arctica*	70	**0.256**	**0.526**	**-0.262**	-0.471	-0.477	-0.189	-0.203	**0.222**	-0.337	**0.554**	
*P*. *pungens*	55	-0.497	-0.482		**0.454**		**0.277**	**0.304**	-0.303	**0.266**	-0.516	
*P*. *seriata* type 1	17		**0.321**	**-0.155**	-0.255	-0.252					**0.283**	**0.18**
*P*. *seriata* type 2	15	-0.314	-0.281			**0.163**					-0.193	
Unk. 161 bp	18				**0.217**					**0.216**	-0.224	
Unk. (208 bp)	7	-0.226	-0.195									
Unk. (170 bp)	6	**0.148**										
Unk. (196 bp)	4	-0.174	-0.137									**0.12**
Unk. (235 bp)	4								**0.151**			
Unk. (240 bp)	3		-0.118		**0.119**							
Unk. (165 bp)	2											

Num, number of occurrences of each taxon in the dataset; Lat, latitude °N; Long, longitude °W; Pres, pressure; Temp, temperature (°C); Sal, salinity; Chl *a*, chlorophyll *a* (mg m^-3^); Phaeo, phaeophytin (mg m^-3^); Trans, transmittance; Fluor, chlorophyll *a* fluorescence; Oxy, dissolved oxygen (mg L^-1^); DA, domoic acid (μg L^-1^).

*Significant positive relationships (p<0.05) are in bold. Rho values > 0.500 are underlined. Blank cells reflect missing data or insignificant relationships.

**Table 5:** Species detected with ARISA in sampled sub-regions and water masses.

Taxon	Sub-Region					Water mass					Other sampling	
	Ber-ing Sea	Ber-ing Strait	Chuk-chi Sea	Bar-row Can-yon	Beau-fort Shelf	ACW	BSW	MWR	NVWW	RWW	ice	flow
Unk-208	**S**		**F**			**F**						**F**
Unk-196	**F**		**F**				**F**					**F**
*P*. *seriata* “type 2”	**F**		**F**			**F**	**F**					**F**
*P*. *pungens*	**F**	**S**	**S/F**	**F**		**S/F**	**S/F**	**S**		**F**		**F**
*P*. *delicatissima*	**F**	**S**	**S/F**	**S/F**	**F**	**S/F**	**S/F**	**S/F**	**F**	**S/F**	**F**	**F**
*P*. *granii*	**F**	**S**	**S/F**	**S/F**	**F**	**S/F**	**S/F**	**S/F**	**F**	**F**	**F**	**F**
*F*. *oceanica*		**S**	**S/F**	**F**	**F**	**S/F**	**S/F**	**S/F**	**S/F**	**S/F**	**F**	
*P*. *obtusa*		**S**	**S/F**	**S/F**	**F**	**S**	**S/F**	**S/F**	**S/F**	**S/F**	**F**	
*P*. *arctica*			**S**	**S/F**	**F**	**S**	**F**	**S/F**	**F**	**S/F**	**F**	
*P*. *seriata* “type 1”				**F**	**F**			**F**	**F**	**F**	**F**	
Unk-235				**S**		**S**		**S**		**S**		
Unk-172				**S**		**S**						
Unk-170				**S/F**		**S**		**S/F**				
Unk-160				**S**				**S**				
Unk-161-162		**S**	**S**	**S**		**S**	**S**	**S**				
Unk-165		**S**		**S**		**S**		**S**				
Unk-240		**S**	**S**			**S**	**S**					
**# of taxa**	**6**	**8**	**11**	**13**	**6**	**14**	**10**	**12**	**6**	**8**	**6**	**6**

S, summer/*Healy* 1801, red shading; F, fall/*Healy* 1803, blue shading; S/F, both summer and fall surveys, yellow shading; Flow, flow-through sample; ACW, Alaskan Coastal Water; BSW, Bering Summer Water; MWR, Melt Water / River Water; NVWW, Newly Ventilated Winter Water; RWW, Remnant Winter Water.

Considering both cruises, sampling spanned ~ 30º longitude and ~ 15º latitude, and 11 taxa were correlated with longitude in the Spearman’s analysis, more than any other parameter. Latitude and longitude were correlated with each other, and negatively correlated with T and S (S5 Table). During the summer cruise, samples from different sub-regions were not statistically different (ANOSIM, p>0.05, S6 Table). The inclusion of the Beaufort Sea during the fall cruise expanded the geographic extent considerably, and assemblages from the Beaufort Sea were statistically different from Bering Sea, Chukchi Sea, and Barrow Canyon assemblages to varying degrees (Table 5), considering both surveys (ANOSIM R = 0.41–0.656, p<0.001) and just the fall survey (R = 0.275–0.322, p<0.001).

The two coldest water masses had fewer observed taxa (6–8) than the warmest ones (12–14; Table 5), and T and S were positively correlated with fluorescence. During the summer cruise, taxa in the RWW stood out as significantly different from those in other water masses, especially ACW (ANOSIM R = 0.629, p<0.001, S6 Table). For the fall cruise, NVWW assemblages differed significantly from all other water masses, especially when compared with ACW (R = 0.955, p<0.001) and BSW (0.899, p<0.001). These and other patterns from the Spearman’s and ANOSIM analyses reflected differences in species composition that were further explored in the PCO analysis, which included T, S, and chlorophyll *a* fluorescence as well as ARISA data for a subset of species.

The PCO analysis was used to help further understand species patterns given seasonal, sub-region, and water mass variability, and explained 66.2% of the data variance (Fig 5). Along the x-axis (dimension 1 = 46.2%), *P*. *delicatissima* (and to a lesser extent *P*. *pungens*) were in opposition to *F*. *oceanica*, *P*. *arctica*, *P*. *seriata* “type 1”, *P*. *granii*, and *P*. *obtusa*. Along the y-axis (dimension 2 = 20%), *F*. *oceanica* (and to a lesser extent *P*. *obtusa*) were in opposition to *P*. *arctica*, *P*. *granii*, and *P*. *seriata* “type 1”. Interestingly, although the short vector established for *P*. *seriata* “type 2” did not stand out as significantly contributing to the observed trends, it was more closely associated with *P*. *pungens* and in opposition to *P*. *seriata* “type 1” (Fig 5). Spearman’s analysis indicated that *P*. *pungens* was positively correlated with *P*. *seriata* “type 2” and they were the few taxa that had positive fall median temperatures (S3 Table). Relative to summer, fall observations for most species included cooler temperature ranges and medians along with more extensive salinity ranges (S3 Table, Fig 5).

The distribution of samples in the PCO analysis depicted a triangle, demonstrating the complexity of teasing apart the overlap between spatial and temporal trends. Samples from the summer cruise were positioned closer to the left vertex, and along the side towards the lower vertex, intermingled with a few samples from the fall cruise (Fig 5A); this configuration reflects the homogeneity already noted through the ANOSIM analysis of taxa along the Bering Strait and the Chukchi Sea (Fig 5B), including the contribution of the warmer water masses more prevalent during, but not exclusive to, the summer cruise (ACW, BSW) and colder water masses during the fall cruise (RWW, NVWW) (Fig 5C). Fall samples also fill out the area in the triangle extending to both vertices to the right, covering samples from the Barrow Canyon and Beaufort Sea regions that were collected in colder water masses across a full range of salinities, from fresher (MWR) to more saline (RWW, NVWW). The x-axis thus reflects the space-time distribution of *P*. *delicatissima* (seconded by *P*. *pungens* and *P*. *seriata* “type 2”) that predominates under conditions of higher water temperatures (especially in the summer cruise, Bering Strait and Chukchi Sea, ACW, MWR) in opposition to the other taxa that prevailed in colder water conditions (especially in the fall cruise, Beaufort Sea, RWW, NVWW). Indeed, the median temperature in which *F*. *oceanica* (-1.40°C), *P*. *arctica* (-1.41°C), and *P*. *granii* (-1.39°C) were found during the fall cruise were the lowest in the whole dataset (S3 Table). The y-axis thus reflects taxa response to a salinity gradient detected between seasons and even within a subregion of the same season (Beaufort Sea in the fall) that sorted out *P*. *arctica*, *P*. *granii*, and *P*. *seriata* “type 1” in less saline waters within MWR as opposed to *F*. *oceanica* and *P*. *obtusa* in more saline conditions within RWW and NVWW.

These observations helped provide context for the shift in species assemblages from the southwest to the northeast, particularly during the fall and with the inclusion of Beaufort Sea samples (Table 5). Recognizing that some sub-regions like the Beaufort Sea were only sampled during one season, only a few overlapping taxa between the Bering Sea and the Beaufort Shelf were observed, namely *P*. *delicatissima* and *P*. *granii*. These species were observed in all sub-regions, all water masses, and in ice and flow samples (Table 5). In spite of their near ubiquitous presence, their contribution to the ARISA varied seasonally and spatially, and *P*. *delicatissima* was more prevalent in the southwestern part of the study region (and comprised >50% of the vast majority of summer ARISA profiles), whereas *P*. *granii* was widespread along the Beaufort Shelf particularly in fall, but still generally comprised <40% of individual ARISA fingerprints (Fig 2).

Differences in the more prevalent species at either edge of the geographic extent were complemented by the presence of other taxa that did not extend across the full spatial domain, but instead appeared to be more polar/northeastern or subpolar/southwestern (Table 5). It is worth pointing out that the six taxa that were observed on the Beaufort Shelf were the same six taxa that occurred in the cold NVWW water mass and in sea ice (Table 5). These appear to represent more polar, Arctic populations and/or species of *Pseudo-nitzschia* [9, 35, 37–40]. Likewise, a separate subset of taxa was observed primarily at lower latitudes and longitudes (i.e., Bering and Chukchi seas), including *P*. *pungens* and *P*. *seriata* “type 2,” that may indicate the contribution of populations and/or species with distribution ranges that extend south to subpolar or temperate latitudes [23, 63].

### IFCB observations

**Fig 6:**
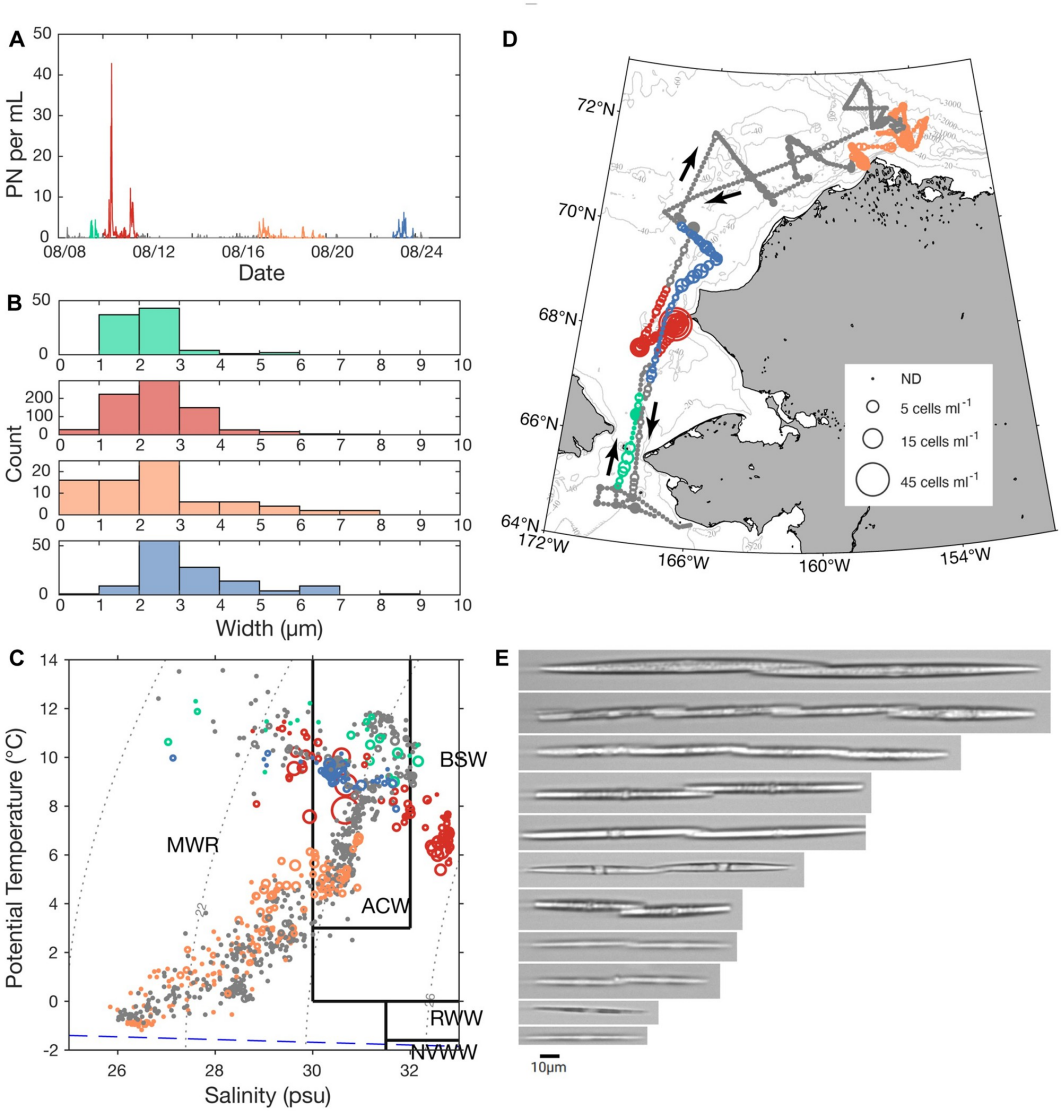
Underway IFCB sampling results for summer (*Healy* 1801). Four patches (differentiated by color) of highest observed *Pseudo-nitzschia* cell abundance are shown: A) *Pseudo-nitzschia* cells mL^-1^ over time; B) size-class distribution of cells based on width in each patch; C) *Pseudo-nitzschia* cellular abundance overlaid on concurrently recorded temperature and salinity measurements; potential density (dotted gray curves), boundaries defining water masses (solid lines) and the freezing point of seawater (blue dashed line) are indicated; D) cellular abundance plotted along the cruise track; arrows indicate direction of travel and asterisk denotes DBO4 transect; E) Examples of cells and chains classified as *Pseudo-nitzschia*.

In situ IFCB sampling along the summer cruise track revealed four distinct patches of *Pseudo-nitzschia* cells based on time of collection (Fig 6A), abundance (Fig 6A–6D), size distribution (Fig 6B), and association with waters masses (Fig 6C) and subregions (Fig 6D). On the outbound transit, a first patch comprised mainly of small cells (1–3 μM in width) was detected in the Bering Strait (max 4 cells mL^-1^, 8/9/18). A second patch, just north of the Bering Strait (along the DB03 transect line) consisted of the highest observed cellular abundances (43 cells mL^-1^, 8/10/18-8/11/18). Cells spanned three water masses (ACW, BSW, and MWR) with highest cell abundances observed in ACW for both patches. Colder, saltier BSW was more prevalent in the second, more northern patch (Fig 6C). The third patch (max 5 cells mL^-1^, 8/16/18-8/20/18), near Barrow Canyon (encompassing DBO5 and a subset of the Outflow Survey), displayed a wide range of cell sizes (up to 9 μm wide), and was associated with ACW and cooler, fresher MWR than the first two patches. The fourth patch (max 6 cells mL^-1^, 8/22/18-8/23/18) encompassed the final transect (Ledyard Bay) on the return transit, primarily coinciding with ACW. Cell size was variable, and the relatively higher cell abundances recorded in the Bering Strait/southern Chukchi Sea and ACW during the outbound transit was not detected on the return track. The size-class used for this analysis may include potential imaging artifacts attributed to the IFCB annotation options and is useful for a relative comparison as opposed to absolute width measurements. To the best of our knowledge, there is not a validly described *Pseudo-nitzschia* species that is 8–9μm wide in valve view; detection of such wide cells were likely specimens in girdle view immediately prior to cell division. While IFCB imagery does not provide enough detail to identify cells to the species level, the variability of cell morphology observed during the cruise (e.g., Fig 6E) validates the diversity and dynamic shifts detected through molecular methods.

### Domoic acid

**Fig 7:**
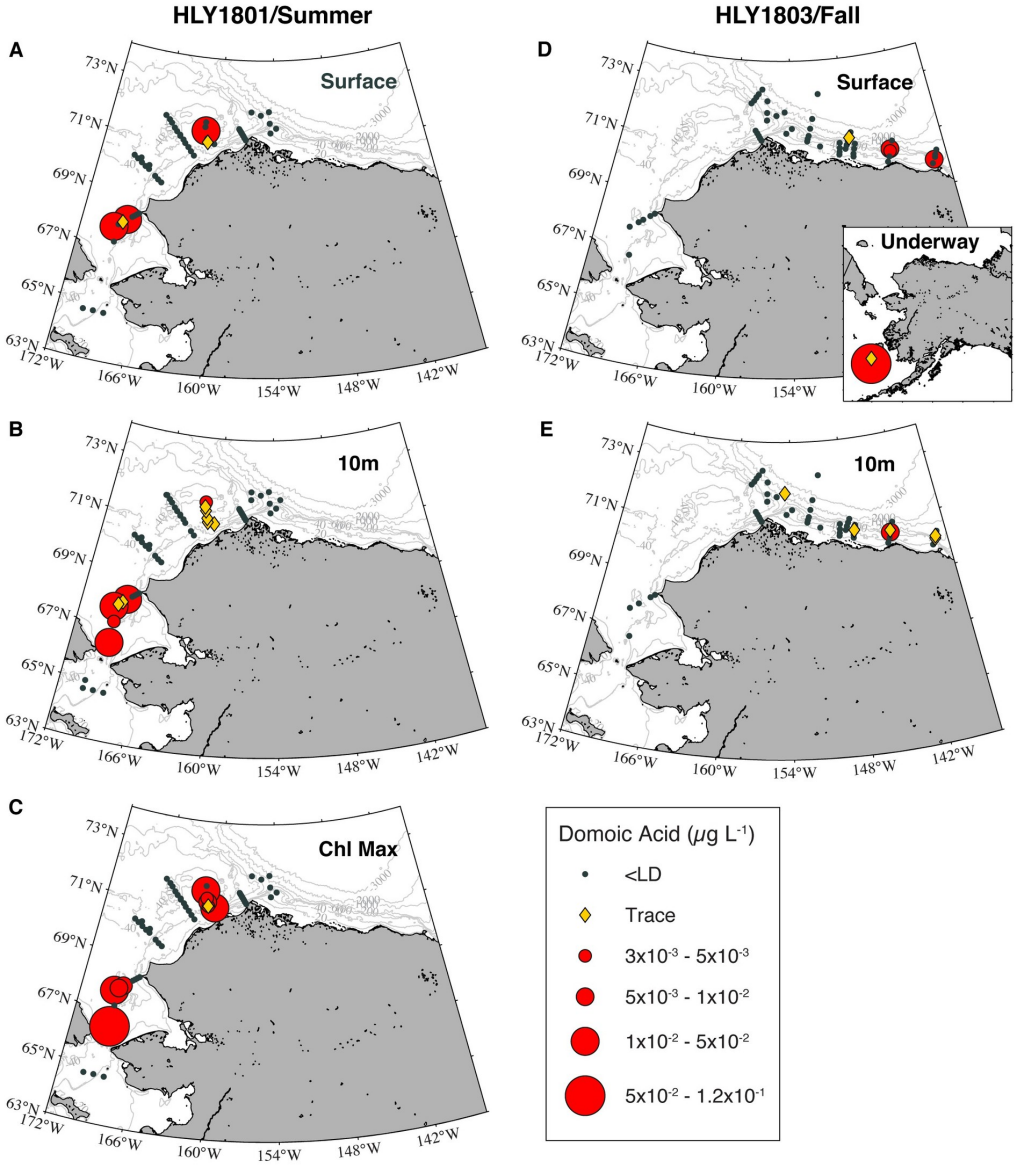
Distribution of particulate domoic acid. Concentrations of DA (ng L^-1^) detected during summer/*Healy* 1801 at surface (A) 10 m (B) and chlorophyll maximum (C) depths, and during fall/*Healy* 1803 at surface (D) and 10 m (E) depths. Inset in panel D displays two underway samples collected on the return transit of the fall cruise.

Approximately 15% of samples tested positive for particulate DA, however, many of these contained only trace levels. Concentrations of DA were negatively correlated with latitude, longitude, and transmissivity, and slightly but positively correlated with depth, salinity, fluorescence and dissolved oxygen; no significant trend was observed with temperature (S5 Table). Five species were significantly correlated with DA (Table 4), and of these, only three were positive (including known toxin producers *P*. *obtusa* and *P*. *seriata* “type 1” and the 196 bp amplicon) and the other two were negative (*P*. *granii* and *P*. *delicatissima*). Examining the spatial patterns of DA (Fig 7) relative to toxic species (Figs 3 and 4) provided helpful insight into the widespread but patchy occurrence of DA during both surveys.

During the summer cruise, DA was observed in 24 samples. Many of these were from the DBO3 and DBO4 transects, with DA concentrations ranging from 4.3 to 130.45 ng L^-1^ (Fig 7A–7C). Based on the IFCB sampling, a localized peak in surface cell abundance occurred along transect line DBO3 but not DBO4. In spite of most summer samples being dominated by *P*. *delicatissima*, these transect lines and in particular the areas where DA was observed had increased proportions of *P*. *obtusa*. For example, ARISA profiles from the DBO4 line were dominated (60–100%) by *P*. *obtusa* and the summer DA maximum of 130.45 ng L^-1^ occurred in a sample from the chl max with an ARISA fingerprint comprised of 92% *P*. *obtusa* and 8% *F*. *oceanica*. That subsurface sample was associated with RWW, however, surface and 10m samples from DBO4 were associated with MWR based on T/S properties. DA was also detected in southern Chukchi Sea samples (stations T-1 and T-2, associated with BSW) at concentrations ranging from 3.0 to 52.3 ng L^-1^. The highest value was found at the chl max, again dominated by *P*. *obtusa* (45%).

During the fall cruise, 12 samples had measurable DA, and those high enough to quantify varied from 3.3 to 111.8 ng L^-1^. Low concentrations (3.3 to 7.4 ng L^-1^) associated with MWR were found in the Beaufort Sea in surface and/or 10m samples on the KTO and the MCK transects (Fig 7D and 7E), where *P*. *seriata* “type 1” was either present or dominant. The highest DA concentration (111.8 ng L^-1^) during the fall cruise was a sample collected in the Bering Sea via the flow-through system, where *P*. *seriata* “type 2” was the dominant species (comprising 45% of the ARISA signal), coincident with *P*. *granii*, *P*. *delicatissima*, *P*. *pungens*, and the 196 and 208 bp taxon, also making it one of the most diverse samples processed.

Three taxa emerged as being associated with DA based on geographic and historical comparisons: *P*. *seriata* “type 1” and “type 2” and *P*. *obtusa*. *P*. *seriata* “type 1” was observed in fall in the Beaufort Sea in MWR, NVWW, and RWW and also in one ice sample. *P*. *seriata* “type 2” was mostly observed around the southern Chukchi Sea in the fall (Figs 2B and 3C), in association with BSW and, to a lesser extent, ACW (Fig 3C). Notably, some of the areas with *P*. *seriata "*type 2” in fall were sampled during summer as well, but did not contain this taxon earlier in the year. The distribution of *P*. *obtusa* varied seasonally and spatially (Figs 2 and 3D), with the greatest proportions designated as colder MWR, RWW and NVWW (Figs 3B and 4C, S3 Table). During summer, *P*. *obtusa* was observed in the Bering Strait, Chukchi Sea, and Barrow Canyon, with highest proportions observed in the Chukchi Sea (DBO4 transect) (Figs 2A and 3B). By fall, *P*. *obtusa* was only really observed in the Barrow Canyon subregion (at DBO5 and OS sites) (Figs 2B and 3B). It is notable that DA occurred across different seasons, regions, and water masses, and in association with multiple *Pseudo-nitzschia* species.

## Discussion

Observations of DA and toxic *Pseudo-nitzschia* species reported here build upon prior reports of DA in phytoplankton [68] and higher trophic levels in subpolar and polar regions of the Arctic [3, 7, 9, 35, 69–71]. These ARISA-based surveys provided sensitive, high-throughput molecular identification of *Pseudo-nitzschia* species composition in >275 water and sea ice samples collected during summer and fall cruises along the Alaska coast in 2018. To consider the relevance of our observations given changing conditions in the Arctic and prior observations in the DBO, 2018 observations relevant to seasonal transitions and interannual variability included: delayed formation and shorter duration of sea ice, sea ice thinning, and reduced sea ice volume [72–74]; spatiotemporal redistribution of primary productivity relative to the prior 15 years [72]; record high sea surface and bottom temperatures in fall [73, 75]; and enhanced inflows of warmer Pacific waters into the region [76]. These conditions impacted the availability and distribution of nutrients as well as phytoplankton and their predators [43, 72, 76] and, collectively, provide an important backdrop for these and future assessments of HAB dynamics. This study of *Pseudo-nitzschia* spp. provides a much needed baseline for evaluating HAB biogeography and trophic transfer of biotoxins in this changing system. The species detected include those previously observed in polar, sub-polar, and temperate regions, as well as two populations of the toxic species *P*. *seriata*, suggesting that *Pseudo-nitzschia* assemblages are poised to respond and adapt to changing conditions on a variety of time scales.

The summer and fall cruises surveyed overlapping and different subregions along the subpolar and polar Alaska coast, with 17 taxa observed in total, half of which were definitively identified using sequencing. Overall, the most commonly observed species during the two 2018 surveys corresponded with *Pseudo-nitzschia* spp. previously identified in Arctic water and/or sea ice in samples from Alaska, Canada, Scandinavia, Greenland, and Russia including *P*. *delicatissima*, *P*. *pungens*, *P*. *seriata*, *P*. *obtusa*, *P*. *granii*, and *P*. *arctica* [9, 35, 65, 77, 78]. Except for *P*. *arctica*, all other *Pseudo-nitzschia* species identified during our study are potential toxin producers [14]. The ARISA method coupled with sequencing allowed us to distinguish two separate *P*. *seriata* types [23] in the Alaska Arctic that were discriminated by a 1-bp insertion and an additional polymorphic nucleotide (sharing 98.3% sequence similarity over 115–116 bp of the ITS1 rRNA).

The allied genus to *Pseudo-nitzschia*, *Fragilariopsis* [79], is among the most abundant marine diatom found in polar regions as planktonic, benthic and/or sea-ice associated species [9, 67, 80–82]. Sequence alignments with the primers used for ARISA (S2 Table) showed the *Pseudo-nitzschia*-specific primers aligned perfectly to *F*. *oceanica*, one of the most commonly reported species at the Pan-Arctic scale, referenced as being ubiquitous in annually-formed sea ice [9]. Indeed, *F*. *oceanica* displayed a widespread spatial distribution beyond the Bering Sea (Table 5) and was present in all ice samples. While the ARISA approach was previously able to successfully amplify a 213 bp fragment from an *F*. *cylindrus* culture [23], this fragment size was not observed during either 2018 cruise and may not amplify well when added to PCRs with species that are a better match to the primers. Similarly, other *Fragilariopsis* species and *Neodenticula seminae*, another common Arctic/sub-Arctic diatom [83], contained additional polymorphisms in the priming sites. These and other issues specific to time series consisting of fragment length analysis highlight the importance of initially and periodically coupling ARISA assessments and more in-depth methods (e.g., ribosomal sequencing, electron microscopy) to most accurately pair ARISA peaks and taxonomic identities.

Hasle (2002) presented the case that *Pseudo-nitzschia* species diversity may be structured according to latitude and/or temperature regime (i.e., originating from warm or cool waters), focusing on 11 *Pseudo-nitzschia* spp., while recognizing that many of the species described at that time were cosmopolites, taxonomic records were still incomplete, and the majority of reports represented intermediate latitudes [84]. Indeed, temperature selection and local adaptation are both thought to be important in structuring diverse phytoplankton species across latitudinal gradients [85]. Of the >57 species of *Pseudo-nitzschia* currently recognized, many are considered cryptic and pseudo-cryptic species complexes for which taxonomic classification utilized a combination of electron microscopy and molecular approaches [14]. While some species still appear to be cosmopolites, others exhibit varying distributions along tropical to polar latitudes, and across estuarine, coastal, and oceanic environments as well as in association with sea ice [9, 14, 19]. Our observations of structured assemblages across latitude/longitude and temperature and salinity regimes—spanning sub-polar and polar waters, from nearshore to offshore, to ice—that include more cosmopolitan species, corroborates Hasle’s earlier insights [84].

While each taxon had distinct spatiotemporal patterns and correlations with environmental parameters, statistical groupings of taxa helped demonstrate biogeographic and ecological distances (Figs 3 and 4; Table 4; S4 and S6 Tables). Clear differences in assemblages were observed (Table 5). The two most commonly observed taxa occurred across all regions (*P*. *delicatissima* and *P*. *granii*), whereas two other taxa occurred everywhere except the Bering Sea (*P*. *obtusa* and *F*. *oceanica*), and one occurred everywhere except for the Beaufort Sea (*P*. *pungens*). The former four taxa and *P*. *arctica* were similarly observed in all water masses and in sea ice samples but displayed unique distribution patterns relative to each other. All other observed taxa, including both *P*. *seriata* types, displayed less ubiquitous distribution (Figs 3 and 4). Further analysis of spatiotemporal distribution and T/S niches provided additional insight into species and DA distribution patterns across seasons and subregions.

The widespread distribution of *P*. *delicatissima* during summer months reflected its broad tolerance and manifested in a general lack of strong differentiation across summer water masses and subregions. There were more substantive shifts in assemblages during fall, across the Chukchi Sea, Barrow Canyon, and the Beaufort Shelf, with more temperate-leaning species such as *P*. *pungens* and *P*. *seriata* “type 2” contributing more significantly to Southern Chukchi Sea samples, while polar-leaning species like *F*. *oceanica*, *P*. *granii*, *P*. *seriata* “type 1”, and *P*. *arctica* made up greater percentages of fall samples taken from the Barrow Canyon and/or Beaufort Shelf regions (Fig 2). To provide more context, in subpolar high nutrient, low chlorophyll (HNLC) Pacific waters, *P*. *granii* has also been recorded along with a number of species not observed in our two surveys including *P*. *fraudulenta*, *P*. cf. *granii*, *P*. *inflatula*, *P*. *heimii*, *P*. *turgidula*, *P*. *pseudodelicatissima*, *P*. cf. *lineola*, and *P*. *dolorosa* [25, 63, 86]. Temperate eastern Pacific *Pseudo-nitzschia* assemblages (from British Columbia to California) include at least 15 species; several overlap with those observed in the present or prior studies in subpolar Pacific or Arctic waters (*P*. *seriata*, *P*. *pseudodelicatissima*, *P*. *delicatissima*, *P*. *pungens*, *P*. *multiseries*, *P*. *inflatula*, *P*. *fraudulenta*) and some have not yet been observed in the Alaskan Arctic (e.g., *P*. *australis*, *P*. *cuspidata*, *P*. *heimii*, *P*. *decipiens*, *P*. *americana*, *P*. *fryxelliana*, *P*. *galaxiae*, and *P*. *hasleana*) based on morphometric and/or genetic taxonomic identification [14, 19, 23, 24, 27, 66]. Since much of the water in the Alaska Arctic originates in the North Pacific, it is likely that there are multiple sources of *Pseudo-nitzschia* to the region, in addition to endemic and/or ice-dwelling species or populations.

Accordingly, water mass associations helped explain these distribution patterns. The majority of unidentified taxa, which were more prevalent in summer but still rarely observed, were present in only certain water masses (mostly ACW, BSW, and/or MWR). Positively correlated with temperature, the temperate species *P*. *pungens* was found in most water masses but was absent from cold, salty NVWW and ice samples, and was not detected in any samples from the Beaufort Shelf. The polar species *P*. *arctica*, detected in all water masses and in the Chukchi Sea, Barrow Canyon and Beaufort Shelf regions, did not occur in the Bering Strait or further south, and was much more prevalent in MWR (with *P*. *seriata* “type 1” and *P*. *granii*). *Pseudo-nitzschia seriata* “type 2” was detected only in ACW and BSW in the Bering Strait and Southern Chukchi subregions, whereas *P*. *seriata* “type 1” was found only in NVWW, MWR, and RWW and mostly in Beaufort Shelf samples; this provides further evidence in support of distinct populations for *P*. *seriata* but the observation of both during fall does not exclude the possibility that these populations co-occur at times. Furthermore, the PCO analysis (Fig 4) reflects taxa structures over a cross-shelf salinity gradient in the Beaufort Sea in the fall (same season and region), with *P*. *arctica*, *P*. *granii*, and *P*. *seriata* “type 1” sorting into less saline waters of the MWR, while *P*. *obtusa* and *F*. *oceanica* occupied more saline, cold, nutrient-rich conditions associated with RWW and NVWW. This is further supported by Spearman’s analysis that confirmed a positive correlation between salinity, and *F*. *oceanica* and *P*. *obtusa* and a negative correlation between salinity and *P*. *arctica*, *P*. *granii*, and *P*. *seriata* “type 1.”

Seasonal variability and species succession may also help explain the variability in DA (see review by Villac et al. 1993 [26, 87]). Notably, DA was observed from the Bering Sea to the Beaufort Sea, in surface waters down to 25.5 m, but was patchy across seasons, subregions and water masses. Summer and fall particulate DA levels (4.3 to 130.45 ng L^-1^) were the same order of magnitude as reported from the Bering Strait in 2017 [68]. Values were one to three orders of magnitude less than those observed by Kvitek et al. (2008) [88] during two *Pseudo-nitzschia* blooms in Monterey Bay where trophic transfer was investigated and peak DA levels were three orders of magnitude greater than maxima reported for marine mammals in Alaska waters by Lefebvre et al. (2016) [3]. While these studies examined different biota and trophic levels, Alaska particulate DA (pDA) measurements collected as part of the present study were not part of an event response, and therefore could reflect conditions that more commonly occur across the region as opposed to peak conditions. In spite of the underlying complexity of factors related to DA production, the presence of potentially high DA-producing species [26–28] indicates that there’s a significant risk of ASP and DA-poisoning in Alaska waters.

The minimally toxic species *P*. *delicatissima* dominated many of the samples collected in the Bering Sea and Barrow Canyon in the summer, yet IFCB data still distinguished four areas of increased *Pseudo-nitzschia* abundance in these regions, a diversity of morphotypes, and an area of high cell density on the outbound transit that was not detected on the return, suggesting dynamics that drive abundance changes over relatively short time scales (i.e., weeks or days). Our data suggest positive correlations between DA and *P*. *obtusa* and *P*. *seriata* “type 1” (Table 4), both of which are large species compared to the small-celled *P*. *delicatissima*. During the summer cruise, DA was found primarily at transects DBO3 north of the Bering Strait and DBO4 in the Chukchi Sea, where *P*. *obtusa* was dominant. Cell abundance determined by the IFCB at two summer DBO3 sites (5 and 8) with DA detected at the surface ranged from 1,675 cells L^-1^ to 2,066 cells L^-1^ allowing us to approximate cellular DA quotas of 11.23 pg cell^-1^ and 6.92 pg cell^-1^, respectively, noting that the intake for the flow-through system and the CTD sampled at slightly different depths. These values fall well above what has been observed for *P*. *obtusa* after induction of toxicity via exposure to copepods (0.2 pg cell^-1^) and within the range reported for Arctic *P*. *seriata* isolates (0.8–33.6 ng L^-1^) and field samples associated with toxigenic *P*. *seriata* [19, 78, 89, 90].

Interestingly, small copepods (*Pseudocalanus*) were observed in sediment traps at sites in the northern Bering Sea and Chukchi Sea coincident with the greatest transport of warmer Pacific waters during 2017–2018 [76]. Grazers can concentrate cells, and could potentially explain the high DA observed in areas such as summer DBO4 samples where DA was observed but the IFCB did not detect cells. Arctic *Calanus* copepodites can not only graze on toxic *Pseudo-nitzschia* species but also seem to induce toxin synthesis and/or increase toxicity or retention in species such as *P*. *obtusa* [78] and *P*. *seriata* [71]. These observations highlight the importance of multitrophic sampling to understand the diverse mechanisms that may drive DA production within Arctic ecosystems.

During the fall cruise, the majority of DA positive samples were found in the Beaufort Sea in water dominated primarily by *P*. *seriata* “type 1”. The highest DA value from the fall cruise, however, was found in a flow-through sample collected south of the Bering Strait that was dominated by *P*. *seriata* “type 2”. The presence but not co-occurrence of both *P*. *seriata* populations during fall was notable not only because this is among the most toxic *Pseudo-nitzschia* species [65, 71, 89], but also for considering potential impacts of changes in the frequency and/or duration of reproductive isolation on future HABs. Furthermore, whether this Arctic population is shared with or distinct from eastern Arctic or North Atlantic ones, and how connected those circulation pathways really are with HAB-forming species survival in mind, could be explored further using population studies. All new Arctic environmental sequences were identical to sequences from isolates corresponding to *P*. *seriata* “type 1” or “type 2”. *Pseudo-nitzschia seriata* “type 1” was previously isolated from the sub-polar and temperate waters of the North Atlantic [38, 89–91] and the Arctic near Greenland [65, 70], and produced DA in culture [89]. *Pseudo-nitzschia seriata* “type 2” was isolated from Puget Sound, Washington, USA and identified using SEM and ITS1 sequencing, but was not tested for toxin production [23].

Indeed, to a certain extent, the water masses defined by T/S that occur in the PAR represent a continuum as Pacific water moves through the Bering Strait, into the Chukchi Sea, and further west. Atlantic water can also manifest in the PAR; however, it typically resides at depth [48] and the appropriate T/S signature was not associated with the samples collected (nor were samples from the deeper parts of the PAR examined). Localized upwelling of Atlantic water on the Beaufort Shelf during November has been previously observed [92] and may provide one mechanism for introduction of deep phytoplankton into photic zones, yet the appropriate T/S signature was not associated with the samples collected (nor were samples from the deeper parts of the PAR examined). Of course, the observation of *P*. *seriata* “type 1” in a sea ice sample suggests that multiple strategies could be involved in the development and maintenance of distinct HAB populations.

The widespread 2015 *Pseudo-nitzschia* bloom along the west coast of the United States [93] and the first observations of *P*. *australis* on the east coast of the US [14, 53] underscore the potential for ocean currents to play an important role in long-distance dispersal of *Pseudo-nitzschia* across latitudinal and/or longitudinal gradients. However, the extent to which this type of dispersal would involve passage through distinct temperature regimes and/or maintenance within a particular temperature regime is not entirely clear [53]. Toxicity may vary on event to interannual time scales based on different mechanisms [14, 94]. Although *P*. *australis* was not confirmed by sequencing samples collected during the current surveys, its presence cannot be ruled out because it shares an ARISA amplicon size of 150 bp (the same as *P*. *seriata* “type 2”). The well documented occurrence of *P*. *australis* blooms in the north Pacific [23, 24, 26, 93] and other temperate species in the PAR suggests that *P*. *australis* similarly has the potential to occur in the Alaska Arctic. The ongoing warming of the polar regions [68] is likely to lead to the geographical expansion of more temperate and potentially toxic species such as *P*. *australis* and *P*. *seriata*, and may lead to life cycle shifts in grazers shown to impact toxicity in *Pseudo-nitzschia* [71, 78, 95]. This heightens concerns about the frequency and persistence of toxic blooms and accumulation of DA in the Arctic food chain [6]. Integrating tools, like the ARISA, IFCB, and LC-MS/MS used in this study, with broader spatiotemporal assessments spanning phytoplankton and higher trophic levels, will be important for connecting patterns of toxicity in phytoplankton and human and wildlife health, now and in the future [6, 96].

## Conclusion

The transitions in *Pseudo-nitzschia* species assemblages, cellular abundance, and DA across broad latitudinal and longitudinal ranges and environmental gradients reveal a diversity of species and potential underlying drivers of biogeographic differences within this genus in polar and sub-polar Alaska waters. The detection of toxic *Pseudo-nitzschia* species in association with DA has important implications for understanding trophic transfer of toxins and threats to human and wildlife health. To varying degrees, toxic *Pseudo-nitzschia* species in this study were found to be significantly correlated with water temperature, salinity, latitude, longitude, subregions, DA, and water masses. It is imperative to determine whether blooms of these potentially toxic species will become more prevalent in changing polar environments and to develop management strategies that minimize human health and ecosystem impacts.

## Supporting information

S1 Fig Sea ice coverage, National Snow and Ice Data Center. The concentration and extent of sea ice, obtained from the National Snow and Ice Data Center (University of Colorado, Boulder) is shown for August, September, October, and November 2018.
(TIF)

S2 Fig Temperature and salinity of stations during 2018 surveys. Stations are plotted (using MATLAB 2021b) in temperature and salinity space, with water mass boundaries designated, for the Summer *Healy* 1801 and Fall *Healy* 1803 cruises, including all collection depths (solid lines show water mass boundaries, dotted lines show potential density, and dashed lines show freezing point of seawater).
(TIF)

S1 Table Summary table showing stations sampled during both the summer (HLY1801) and the fall (HLY1803) cruises. The latitude, longitude, date sampled, target depth and parameters monitored are listed. The actual sample depths are listed in the cruise data repository. Note that these parameters were not monitored for ice samples, flow through samples and a small subset of samples from HLY1803.
(DOCX)

S2 Table Pnall primer specificity relative to *Fragilariopsis* spp. The number of mismatches are indicated for each *Fragilariopsis* species, as is the location of the mismatches to the forward *Pseudo-nitzschia* specific (Pnall) primers. The Genbank accession number for each *Fragilariopsis* sequence is listed, as is the location of origin.
(DOCX)

S3 Table Species ranges from ARISA data. The conditions (temperature, salinity, extracted chlorophyll, and dissolved oxygen), and geographic range (based on latitude and longitude), of the eight most commonly observed taxa during each survey based on ARISA data. The minimum value observed (min), maximum value observed (max), and median value observed (mdn) are indicated for conditions, and the geographic range used maximum and minimum values from the present study.
(DOCX)

S4 Table Summary of Spearman’s correlation coefficients (rho) between most commonly observed taxa. Significant relationships (p<0.05) are in bold. Rho values ≥ 0.5 are underlined.
(DOCX)

S5 Table Summary of Spearman’s correlation coefficients (rho) between various environmental factors. Significant relationships (p<0.05) are in bold. Rho values ≥ 0.5 are underlined.
(DOCX)

S6 Table Results for the ANOSIM permutation-based hypothesis testing. The ANOSIM test was applied to surveys (*Healy* 1801 in summer and/or *Healy* 1803 in fall), subregions (Bering Strait, Chukchi Sea, Barrow Canyon, Beaufort Sea) and water masses. Significant level of sample statistic is 0.1% (marked in bold).
(DOCX)
