# Supplementary figures and images for: Spatiotemporal transitions in Pseudo-nitzschia species assemblages and domoic acid along the Alaska coast
Source: PLoS One. 2023 Mar 22;18(3):e0282794. doi: 10.1371/journal.pone.0282794 (PMC10032537; doi:10.1371/journal.pone.0282794)

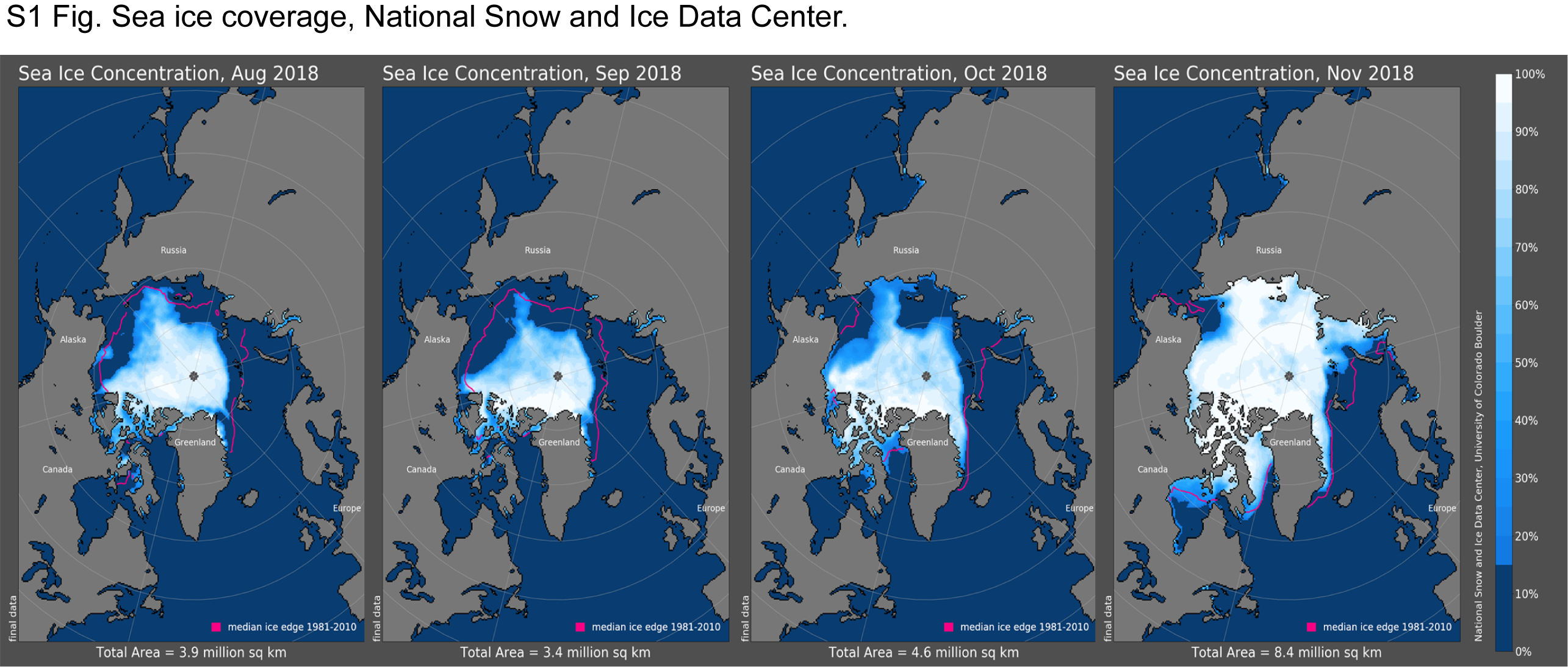

Supplement: S1 Fig — The concentration and extent of sea ice, obtained from the National Snow and Ice Data Center (University of Colorado, Boulder) is shown for August, September, October, and November 2018. (TIF) [file pone.0282794.s001.tif]

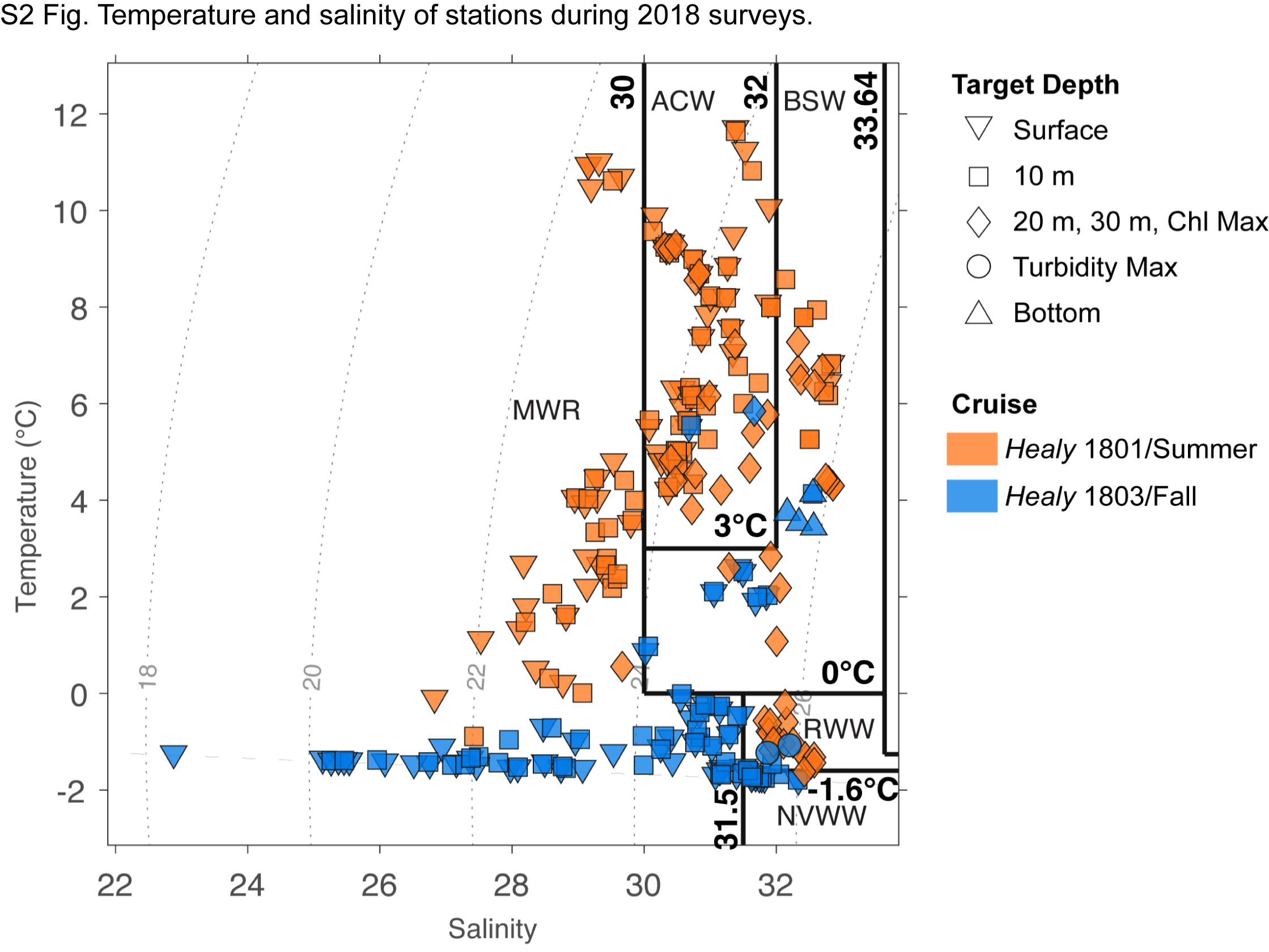

Supplement: S2 Fig — Stations are plotted (using MATLAB 2021b) in temperature and salinity space, with water mass boundaries designated, for the Summer Healy 1801 and Fall Healy 1803 cruises, including all collection depths (solid lines show water mass boundaries, dotted lines show potential density, and dashed lines show freezing point of seawater). (TIF) [file pone.0282794.s002.tif]
