# Supplementary material for: Spatiotemporal transitions in Pseudo-nitzschia species assemblages and domoic acid along the Alaska coast
Source: PLoS One. 2023 Mar 22;18(3):e0282794. doi: 10.1371/journal.pone.0282794 (PMC10032537; doi:10.1371/journal.pone.0282794)
Supplement: S1 Table — The latitude, longitude, date sampled, target depth and parameters monitored are listed. The actual sample depths are listed in the cruise data repository. Note that these parameters were not monitored for ice samples, flow through samples and a small subset of samples from HLY1803. (DOCX) [file pone.0282794.s003.docx]

**S1 Table.** **Summary table showing stations sampled during both the summer (HLY1801) and the fall (HLY1803) cruises.** The latitude, longitude, date sampled, target depth and parameters monitored are listed. Note that these parameters were not monitored for ice samples, flow through samples and a small subset of samples from HLY1803. Actual sample depths are listed in the cruise data repository.

|  |  |  | **summer -1801** | | | **fall -1803** | | |
| --- | --- | --- | --- | --- | --- | --- | --- | --- |
| **Station Name** | **Latitude**  **(N)** | **Longitude**  **(W)** | **Date** | **Target Depth** | **Para-meters^*, **^** | **Date** | **Target Depth*** | **Param-eters^*, **^** |
| C-2 | 71.2327 | -164.2207 | 8/13 | S, 10, C | All | - | - | - |
| C-3 | 71.8297 | -166.0737 | 8/12 | S, 10, C | All | - | - | - |
| C-4 | 71.0483 | -160.4822 | 8/15 | S, 10, C | All | - | - | - |
| C-10 | 70.2197 | -167.7935 | 8/12 | S, 10, C | All | - | - | - |
| C-11 | 70.0162 | -166.855 | 8/11 | S, 10, C | All | - | - | - |
| DBO2-1 | 64.6718 | -169.9258 | 8/9 | S, 10, C | All | - | - | - |
| DBO2-2 | 64.68 | -169.1002 | 8/9 | S, 10 | All | - | - | - |
| DBO2-3 | 64.6705 | -168.2342 | 8/8 | S, 10, C | All | - | - | - |
| DBO2-4 | 64.9598 | -169.899 | 8/9 | 10 | All | - | - | - |
| DBO3-1 | 68.3012 | -166.9308 | 8/10 | S, 10 | All | 11/15 | S, 10 | -chl^*^ |
| DBO3-2 | 68.2462 | -167.1255 | 8/10 | S, 10, C | All | - | - | - |
| DBO3-3 | 68.1902 | -167.3027 | 8/10 | S, 10, C | All | 11/15 | S, 10, B | -chl |
| DBO3-4 | 68.1357 | -167.4915 | 8/10 | S, 10 | All | - | - | - |
| DBO3-5 | 68.0153 | -167.8798 | 8/10 | S, 10, C | All | 11/15 | S, 10, B | -chl |
| DBO3-6 | 67.8968 | -168.248 | 8/10 | S, 10, C | All | 11/15 | S, 10 | -chl |
| DBO3-7 | 67.7853 | -168.601 | 8/11 | S, 10, C | All | - | - | - |
| DBO3-8 | 67.677 | -168.9568 | 8/11 | S, 10, C | All | 11/15 | S, 10, B | -chl |
| DBO4-1N | 71.09 | -161.1928 | 8/15 | S, 10, C | All | - | - | - |
| DBO4-2N | 71.2238 | -161.2893 | 8/15 | S, 10, C | All | - | - | - |
| DBO4-3N | 71.3507 | -161.3958 | 8/15 | C | All | - | - | - |
| DBO4-4N | 71.4805 | -161.5048 | 8/15 | S, 10, C | All | - | - | - |
| DBO4-5N | 71.6103 | -161.6152 | 8/15 | S, 10, C | All | - | - | - |
| DBO4-6N | 71.7755 | -161.5865 | 8/16 | S, 10, C | All | - | - | - |
| DBO5-1 | 71.2468 | -157.135 | 8/17 | S, 10 | All | 11/14 | S, 10 | -chl |
| DBO5-2 | 71.2888 | -157.2213 | 8/17 | S, 10, 30 | All | - | - | - |
| DBO5-3 | 71.3262 | -157.3067 | 8/17 | S, 10 | All | 11/14 | S, 10 | -chl |
| DBO5-4 | 71.3727 | -157.3803 | 8/17 | S, 10 | All | - | - | - |
| DBO5-5 | 71.4095 | -157.4497 | 8/17 | S, 10 | All | 11/14 | S, 10 | -chl |
| DBO5-6 | 71.4543 | -157.5532 | 8/17 | S, 10 | All | - | - | - |
| DBO5-7 | 71.4953 | -157.6265 | 8/17 | S, 10 | All | 11/14 | S, 10 | -chl |
| DBO5-8 | 71.5358 | -157.7107 | 8/17 | S, 10, C | All | - | - | - |
| DBO5-9 | 71.5835 | -157.8175 | 8/17 | S, 10 | All | 11/14 | S, 10 | -chl |
| DBO5-10 | 71.6262 | -157.9005 | 8/17 | S, 10 | All | - | - | - |
| DBO6-2 | 71.2092 | -152.2177 | - | - | - | 10/30 | S, 10 | -chl |
| DBO6-6 | 71.3997 | -152.0352 | - | - | - | 10/30 | S, 10 | -chl |
| DBO6-4 | 71.2967 | -152.148 | - | - | - | 10/30 | S, 10 | -chl |
| DBO6-12 | 71.6463 | -151.8288 | - | - | - | 10/31 | S, 10 | -chl |
| DBO6-15 | 71.9805 | -151.5428 | - | - | - | 10/31 | S, 10 | -chl |
| DBO6-18 | 72.8283 | -150.7468 | - | - | - | 10/31 | S, 10 | -chl |
| IC-1 | 71.8302 | -165.9698 | 8/12 | 10, C | All | - | - | - |
| IC-10 | 70.7172 | -162.8565 | 8/14 | S, 10, C | All | - | - | - |
| IC-11 | 70.58 | -162.4912 | 8/14 | 10, C | All | - | - | - |
| IC-2 | 71.7052 | -165.6028 | 8/12 | S, 10, C | All | - | - | - |
| IC-3 | 71.6005 | -165.3038 | 8/13 | S, 10, C | All | - | - | - |
| IC-4 | 71.4485 | -164.919 | 8/13 | S, 10, C | All | - | - | - |
| IC-5 | 71.3367 | -164.6127 | 8/13 | S, 10, C | All | - | - | - |
| IC-6 | 71.195 | -164.2018 | 8/13 | S, 10, C | All | - | - | - |
| IC-7 | 71.0847 | -163.8018 | 8/13 | S, 10, C | All | - | - | - |
| IC-8 | 70.9725 | -163.5642 | 8/14 | S, 10, C | All | - | - | - |
| IC-9 | 70.849 | -163.1872 | 8/14 | S, C | All | - | - | - |
| LB-10 | 69.9567 | -167.2213 | 8/23 | S, 10, 20 | All | - | - | - |
| LB-11 | 70.0578 | -167.6565 | 8/22 | S, 10, 30 | All | - | - | - |
| LB-12 | 70.1648 | -168.1307 | 8/22 | S, 10, 20 | All | - | - | - |
| LB-13 | 70.2598 | -168.5445 | 8/22 | S, 10, C | All | - | - | - |
| LB-5 | 69.4962 | -165.3785 | 8/23 | S, 10, 20 | All | - | - | - |
| LB-6 | 69.5815 | -165.7402 | 8/23 | S, 10, 20 | All | - | - | - |
| LB-7 | 69.6832 | -166.0928 | 8/23 | S, 10, 20 | All | - | - | - |
| LB-9 | 69.879 | -166.8215 | 8/23 | S, 10, 20 | All | - | - | - |
| OS1-2 | 71.9475 | -156.5995 | 8/20 | 10 | -chl | - | - | - |
| OS1-6 | 72.2178 | -156.5987 | 8/20 | S, 10 | -chl | - | - | - |
| OS1-12 | 72.6198 | -156.6018 | 8/21 | C | -chl | - | - | - |
| OS2-6 | 72.1093 | -155.54 | 8/20 | S, 10, C | -chl | - | - | - |
| OS3-4 | 71.496 | -154.4728 | 8/18 | S, 10 | -chl | - | - | - |
| OS3-9 | 71.833 | -154.5462 | 8/19 | S, 10 | -chl | - | - | - |
| OS3-15 | 72.2347 | -154.6282 | 8/19 | S, 10, C | -chl | - | - | - |
| OS4-2 | 71.95 | -153.3597 | 8/18 | 10, C | All | - | - | - |
| OS4-7 | 71.6567 | -153.8437 | 8/18 | S, 10, C | All | - | - | - |
| T-1 | 66.417 | -168.6775 | 8/9 | 10, C | All | - | - | - |
| T-2 | 67.1642 | -168.6635 | 8/9 | S, 10, C | All | - | - | - |
| BW1-1 | 71.0128 | -150.8692 | - | - | - | 11/12 | S, 10, I | -chl |
| KTO-1 | 70.2007 | -144.0215 | - | - | - | 11/5 | S, 10 | -chl |
| KTO-3 | 70.3712 | -143.7857 | - | - | - | 11/5 | S, 10 | -chl |
| KTO-5 | 70.555 | -143.6148 | - | - | - | 11/5 | S, 10 | -chl |
| KTO-6 | 70.6248 | -143.5888 | - | - | - | 11/5 | S, 10 | -chl |
| KTO-9 | 70.8967 | -143.2197 | - | - | - | 11/6 | S, 10 | -chl |
| MCK-1 | 69.8165 | -139.61 | - | - | - | 11/4 | S, 10 | -chl |
| MCK-3 | 69.9393 | -139.3863 | - | - | - | 11/4 | S, 10 | -chl |
| MCK-5 | 70.012 | -139.2382 | - | - | - | 11/4 | S, 10 | -chl |
| MCK-7 | 70.0918 | -139.1635 | - | - | - | 11/5 | S, 10 | -chl |
| MCK-9 | 70.2457 | -138.926 | - | - | - | 11/5 | S, 10 | -chl |
| OS1-2 | 71.9475 | -156.5995 | - | - | - | 11/8 | S, 10, T | -chl |
| OS1-7 | 71.6588 | -153.8363 | - | - | - | 11/8 | S, 10, T | -chl |
| OS2-1 | 71.496 | -154.4557 | - | - | - | 11/9 | S, 10 | -chl |
| OS2-6 | 72.1093 | -155.54 | - | - | - | 11/9 | S, 10 | -chl |
| OS2-12 | 72.2325 | -154.6262 | - | - | - | 11/9 | S, 10, I | -chl |
| OS3-6 | 72.1138 | -155.5573 | - | - | - | 11/10 | S, 10 | -chl |
| OS4-1 | 71.9515 | -156.5838 | - | - | - | 11/10 | S, 10 | -chl |
| OS4-5 | 72.2103 | -156.6012 | - | - | - | 11/10 | S, 10 | -chl |
| OS4-11 | 72.617 | -156.601 | - | - | - | 11/10 | S, 10 | -chl |
| OS5-2 | 73.0149 | -157.238 | - | - | - | 11/10 | S, 10, I | -chl |
| OS5-5 | 72.8515 | -157.6512 | - | - | - | 11/11 | S, 10, I | -chl |
| OS5-7 | 72.7408 | -157.909 | - | - | - | 11/11 | S, 10, I | -chl |
| OS5-9 | 72.623 | -158.1457 | - | - | - | 11/11 | S, 10, I | -chl |
| OS5-11 | 72.5222 | -158.4387 | - | - | - | 11/11 | S, 10, I | -chl |
| PRE-1 | 70.9932 | -147.1588 | - | - | - | 11/7 | S, 10 | -chl |
| PRE-5 | 70.8207 | -147.306 | - | - | - | 11/7 | S, 10 | -chl |
| PRE-9 | 70.6482 | -147.4445 | - | - | - | 11/7 | S, 10 | -chl |
| PRE-11 | 70.5552 | -147.4647 | - | - | - | 11/7 | S, 10 | -chl |
| PRB-3 | 70.8552 | -148.2032 | - | - | - | 11/2 | S, 10 | -chl |
| PRB-6 | 70.9778 | -148.0382 | - | - | - | 11/2 | S, 10 | -chl |
| PRB-9 | 71.0985 | -147.8785 | - | - | - | 11/3 | S, 10 | -chl |
| PRB-12 | 71.223 | -147.6932 | - | - | - | 11/3 | S, 10 | -chl |
| PRB-15 | 71.4053 | -147.4588 | - | - | - | 11/3 | S | -chl |
| PRW-1 | 70.679 | -148.9078 | - | - | - | 11/7 | S, 10, I | -chl |
| PRW-3 | 70.7698 | -148.8665 | - | - | - | 11/7 | S, 10 | -chl |
| PRW-6 | 70.9023 | -148.8033 | - | - | - | 11/7 | S, 10 | -chl |
| PRW-10 | 71.0805 | -148.7543 | - | - | - | 11/8 | S, 10 | -chl |
| Test Cast | 66.7235 | -168.4602 | - | - | - | 10/28 | S, 10, C | All |
| Transit-3 | 57.7853 | -167.6017 | - | - | - | 10/26 | FT | - |
| Transit-4 | 58.3853 | -167.7836 | - | - | - | 10/27 | FT | - |
| XBT-4 | 70.5858 | -146.1706 | - | - | - | 11/6 | I | - |

S, surface; CM, vertical chlorophyll maximum; Btm, bottom; TM, vertical turbidity max, I, ice.

^*^All=temperature, salinity, dissolved oxygen, chlorophyll *a* (extracted and in situ fluorescence), and ARISA data.

^**^-chl = all parameters listed above except extracted chlorophyll *a* data.
