# Supplementary material for: Spatiotemporal transitions in Pseudo-nitzschia species assemblages and domoic acid along the Alaska coast
Source: PLoS One. 2023 Mar 22;18(3):e0282794. doi: 10.1371/journal.pone.0282794 (PMC10032537; doi:10.1371/journal.pone.0282794)
Supplement: S2 Table — The number of mismatches are indicated for each Fragilariopsis species, as is the location of the mismatches to the forward Pseudo-nitzschia specific (Pnall) primers. The Genbank accession number for each Fragilariopsis sequence is listed, as is the location of origin. (DOCX) [file pone.0282794.s004.docx]

**S2 Table.** **Pnall primer specificity relative to *Fragilariopsis* spp.** The number of mismatches are indicated for each *Fragilariopsis* species, as is the location of the mismatches to the *Pseudo-nitzschia* specific (Pnall) primers. The Genbank accession number for each *Fragilariopsis* sequence is listed, as is the location of origin.

| **Species/type** | **Predicted ARISA length (bp)** | **Primer Mismatches** | **Origin** | **GenBank accession no.** |
| --- | --- | --- | --- | --- |
| *Fragilariopsis oceanica* | 190 | 0 | Canadian Arctic, Beaufort Sea  71°4.741'N, 133°38.872'W | GU170661.1 |
| *Fragilariopsis reginae-jahniae* | 191 | 1(F) | Gulf of St. Lawrence, Anticosti gyre  49°43'N, 66°15'W | GU170663.1 |
| *Fragilariopsis rhombica* | 228 | 3(F) | Ross Sea,  66°58'63” S, 149°58'53”W | GU734803.1 |
| *Fragilariopsis curta* | 228 | 3(F) | Ross Sea, Ice  74°0'2” S, 140°0'19”W | GU170666.1 |
| *Fragilariopsis kerguelensis* | 228 | 3(F) | Ross Sea,  65°25'70” S, 149°58'1”W | EF660061.1 |
| *Fragilariopsis*  *vanheurckii* | 229 | 3(F) | Ross Sea, Ice  74°59'S, 140° 0' 19”W | GU170667.1 |
| *Fragilariopsis nana* | 210 | 2(F) | Weddell Sea, Ice | EF660059.1 |
| *Fragilariopsis*  *cylindrus* | 211 | 2(F) | Gulf of St. Lawrence, Anticosti gyre  49°43'N, 66°15'W | EF660055.1 |
| *Fragilariopsis*  *doliolus* | 213 | 2(F), 1(R) | Indian Ocean, TARA_066 | KY782385.1 |
| *Fragilariopsis*  *pacifica* | 207 | 3(F), 2(R) | Twin Harbor, Washington State | GU170665.1 |
| *Neodenticula seminae* | 208 | 2(F) | Gulf of St. Lawrence 49.7°N, 66.25°W;  Subarctic Pacific Station SA 49°N, 174°W | GU734799.1,  GU734800.1 |

Bp, base pairs; F, forward primer; R, reverse primer
