# Supplementary material for: Spatiotemporal transitions in Pseudo-nitzschia species assemblages and domoic acid along the Alaska coast
Source: PLoS One. 2023 Mar 22;18(3):e0282794. doi: 10.1371/journal.pone.0282794 (PMC10032537; doi:10.1371/journal.pone.0282794)
Supplement: S3 Table — The conditions (temperature, salinity, extracted chlorophyll, and dissolved oxygen), and geographic range (based on latitude and longitude), of the eight most commonly observed taxa during each survey based on ARISA data. The minimum value observed (min), maximum value observed (max), and median value observed (mdn) are indicated for conditions, and the geographic range used maximum and minimum values from the present study. (DOCX) [file pone.0282794.s005.docx]

**S3 Table. Species ranges from ARISA data.** The conditions (temperature, salinity, extracted chlorophyll, and dissolved oxygen), and geographic range (based on latitude and longitude), of the eight most commonly observed taxa during each survey based on ARISA data. The minimum value observed (min), maximum value observed (max), and median value observed (mdn) are indicated for conditions, and the geographic range used maximum and minimum values from the present study.

| ***HEALY* 1801**  **(summer)** | **Temperature (°C)** | | | | **Salinity (psu)** | | | **Chlorophyll (mg m^-3^)** | | | **Oxygen (mg L^-1^)** | | | **Geo. Range** | | |
| --- | --- | --- | --- | --- | --- | --- | --- | --- | --- | --- | --- | --- | --- | --- | --- | --- |
|  | **min** | **max** | | **mdn** | **min** | **max** | **mdn** | **min** | **max** | **mdn** | **min** | **max** | **mdn** | **Lat.** | **Long.** | |
| ***P. delicatissima*** (n=163) | -1.42 | 11.70 | | 5.56 | 26.81 | 32.86 | 30.73 | 0.18 | 15.19 | 0.84 | 5.96 | 9.73 | 7.13 | 64.67- 72.62 | -169.93-  -153.36 | |
| ***P. obtusa*** (n=53) | -1.62 | 9.49 | | 3.53 | 28.36 | 32.79 | 31.36 | 0.18 | 15.19 | 0.67 | 5.96 | 11.20 | 7.60 | 66.42-  72.23 | -168.96-  -153.84 | |
| ***F. oceanica*** (n=34) | -1.62 | 9.49 | | 2.34 | 28.81 | 32.86 | 32.01 | 0.20 | 15.19 | 1.02 | 5.96 | 10.10 | 7.22 | 66.42-  71.83 | -168.96-  -160.48 | |
| ***P. pungens*** (n=37) | 1.61 | 11.02 | | 9.00 | 28.82 | 32.42 | 30.53 | 0.29 | 2.40 | 1.15 | 6.13 | 8.26 | 6.52 | 67.16-  71.48 | -168.66-  -161.50 | |
| ***P. granii*** (n=19) | -0.90 | 6.73 | | 3.53 | 26.81 | 32.70 | 29.79 | 0.18 | 15.19 | 0.47 | 6.36 | 8.64 | 7.73 | 64.96-  72.23 | -169.90-  -153.36 | |
| ***P. arctica*** (n=10) | -1.42 | 9.20 | | 2.72 | 26.81 | 32.48 | 29.64 | 0.18 | 1.63 | 0.41 | 6.53 | 9.58 | 7.86 | 69.68-  71.95 | -166.09-  -153.36 | |
| **unknown (170 bp)** (n=5) | 0.56 | 5.66 | | 3.81 | 28.11 | 30.73 | 30.08 | 0.46 | 0.97 | 0.64 | 6.97 | 8.70 | 7.52 | 71.33-  72.62 | -157.82-  -153.36 | |
| **unknown (161 bp)** (n=18) | 0.56 | 9.33 | | 5.40 | 29.24 | 32.51 | 30.50 | 0.46 | 2.30 | 0.96 | 6.13 | 8.70 | 6.91 | 64.96-  71.95 | -169.90-  -153.36 | |
| ***HEALY* 1803**  **(fall)** | **Temperature (°C)** | | | | **Salinity (psu)** | | | **Chlorophyll (mg m^-3^)** | | | **Oxygen (mg L^-1^)** | | | **Geo. Range** | | |
|  | **min** | **max** | **mdn** | | **min** | **max** | **mdn** | **min** | **max** | **mdn** | **min** | **max** | **mdn** | **Lat.** | **Long.** | |
| ***P. delicatissima*** (n=92) | -1.75 | 5.85 | -1.00 | | 26.75 | 32.57 | 31.06 | n/a | n/a | n/a | 5.50 | 8.84 | 8.15 | 57.79-  73.01 | | -168.96-  -147.16 |
| ***P. obtusa*** (n=23) | -1.72 | 4.13 | -1.16 | | 27.99 | 32.57 | 31.34 | n/a | n/a | n/a | 5.85 | 8.37 | 8.00 | 67.68-  72.62 | | -168.96-  -139.61 |
| ***F. oceanica*** (n=63) | -1.79 | 5.85 | -1.40 | | 25.26 | 32.57 | 31.61 | n/a | n/a | n/a | 5.50 | 8.99 | 8.18 | 66.72-  73.01 | | -168.96-  -138.93 |
| ***P. pungens*** (n=18) | -1.46 | 5.85 | 2.61 | | 30.67 | 32.57 | 31.68 | n/a | n/a | n/a | 5.50 | 7.92 | 7.26 | 57.79-  71.25 | | -168.96-  -157.14 |
| ***P. granii*** (n=89) | -1.75 | 5.52 | -1.39 | | 25.46 | 32.06 | 30.81 | n/a | n/a | n/a | 6.86 | 8.95 | 8.35 | 57.79-  73.01 | | -168.96-  -139.24 |
| ***P. arctica*** (n=60) | -1.74 | 0.98 | -1.41 | | 22.88 | 32.28 | 28.61 | n/a | n/a | n/a | 7.92 | 9.02 | 8.67 | 69.82-  73.01 | | -158.44-  -138.93 |
| ***P. seriata* “type 2”** (n=15) | 1.99 | 5.85 | 3.44 | | 30.67 | 32.57 | 31.50 | n/a | n/a | n/a | 5.50 | 7.63 | 6.86 | 57.79-  68.30 | | -166.93-  -168.96 |
| ***P. seriata* “type 1”** (n=17) | -1.70 | 0.00 | -1.37 | | 22.88 | 31.65 | 27.44 | n/a | n/a | n/a | 8.01 | 9.02 | 8.71 | 69.94-  72.11 | | -156.58-  -139.39 |
| **unknown (208 bp)** (n=7) | 1.91 | 5.52 | 2.52 | | 30.67 | 31.87 | 31.50 | n/a | n/a | n/a | 6.86 | 7.43 | 7.34 | 57.79-  68.19 | | -168.96-  -167.30 |
