# Supplementary material for: Spatiotemporal transitions in Pseudo-nitzschia species assemblages and domoic acid along the Alaska coast
Source: PLoS One. 2023 Mar 22;18(3):e0282794. doi: 10.1371/journal.pone.0282794 (PMC10032537; doi:10.1371/journal.pone.0282794)
Supplement: S4 Table — Significant relationships (p<0.05) are in bold. Rho values ≥ 0.5 are underlined. (DOCX) [file pone.0282794.s006.docx]

**Table S4.** **Summary of Spearman’s correlation coefficients (rho) between most commonly observed taxa.** Significant relationships (p<0.05) are in bold. Rho values ≥ 0.5 are underlined.

|  | *F. oceanica* | *P. granii* | *P. arctica* | *P. obtusa* | *P. pungens* | *P. seriata* "type 2" | *P. seriata* "type 1" | unknown (161 bp) | unknown (208 bp) | unknown (170 bp) | unknown (196 bp) | unknown (235 bp) | unknown (240 bp) | unknown (172 bp) |
| --- | --- | --- | --- | --- | --- | --- | --- | --- | --- | --- | --- | --- | --- | --- |
| *P. delicatissima* | -0.55 | -0.32 | -0.458 | -0.21 |  | -0.163 | -0.306 | **0.216** |  |  |  |  |  |  |
| *F. oceanica* |  |  |  | **0.152** |  | **0.13** |  | -0.172 |  |  |  |  |  |  |
| *P. granii* |  |  | **0.449** | -0.142 | -0.277 |  |  | -0.164 |  |  |  |  |  |  |
| *P. arctica* |  |  |  | -0.136 | -0.226 | -0.126 | **0.329** | -0.139 |  |  |  |  |  |  |
| *P. obtusa* |  |  |  |  |  |  | -0.117 | -0.147 |  |  |  | **0.209** |  |  |
| *P. pungens* |  |  |  |  |  | **0.454** | -0.117 |  | **0.205** |  | **0.171** |  | **0.116** |  |
| *P. seriata* "type 2" |  |  |  |  |  |  |  |  | **0.475** |  | **0.512** |  |  |  |
| *P. seriata* "type 1" |  |  |  |  |  |  |  |  |  |  |  |  |  |  |
| unknown (161 bp) |  |  |  |  |  |  |  |  |  | **0.171** |  |  |  |  |
| unknown (208 bp) |  |  |  |  |  |  |  |  |  |  | **0.375** |  |  |  |
| unknown (170 bp) |  |  |  |  |  |  |  |  |  |  |  |  |  | **0.287** |

Unk, unknown; bp, base pairs.
