# Supplementary material for: Spatiotemporal transitions in Pseudo-nitzschia species assemblages and domoic acid along the Alaska coast
Source: PLoS One. 2023 Mar 22;18(3):e0282794. doi: 10.1371/journal.pone.0282794 (PMC10032537; doi:10.1371/journal.pone.0282794)
Supplement: S5 Table — Significant relationships (p<0.05) are in bold. Rho values ≥ 0.5 are underlined. (DOCX) [file pone.0282794.s007.docx]

**Table S5. Summary of Spearman’s correlation coefficients (rho) between various environmental factors.** Significant relationships (p<0.05) are in bold. Rho values ≥ 0.5 are underlined. Abbreviations for environmental parameters:

|  | Long | Depth | Pres | Temp | Sal | Chl *a* | Phaeo | Trans | Fluor | Oxy | DA |
| --- | --- | --- | --- | --- | --- | --- | --- | --- | --- | --- | --- |
| Lat | **0.532** | -0.127 |  | -0.45 | -0.448 | -0.453 | -0.47 | **0.552** | -0.361 | **0.455** | -0.223 |
| Long |  | -0.333 | -0.324 | -0.768 | -0.464 | -0.367 | -0.42 | **0.427** | -0.493 | **0.675** | -0.141 |
| Depth |  |  | **0.989** | **0.144** | **0.44** | **0.408** | **0.466** | -0.359 | **0.408** | -0.251 | **0.161** |
| Pres |  |  |  | **0.147** | **0.441** | **0.433** | **0.487** | -0.382 | **0.408** | -0.251 | **0.158** |
| Temp |  |  |  |  |  | **0.181** | **0.203** | -0.219 | **0.522** | -0.777 |  |
| Sal |  |  |  |  |  | **0.478** | **0.503** | -0.503 | **0.237** | -0.289 | **0.164** |
| Chl *a* |  |  |  |  |  |  | **0.859** | -0.71 | **0.825** | -0.161 | **0.323** |
| Phaeo |  |  |  |  |  |  |  | -0.706 | **0.702** | -0.278 |  |
| Trans |  |  |  |  |  |  |  |  | -0.693 | 0.424 | -0.251 |
| Fluor |  |  |  |  |  |  |  |  |  | -0.42 | **0.246** |
| Oxy |  |  |  |  |  |  |  |  |  |  | **0.243** |

Temp, temperature; Sal, salinity; Oxy, dissolved oxygen; Fluor, chlorophyll *a* fluorescence; Trans, transmittance; Chl *a*, extracted chlorophyll *a*; Phaeo, phaeophytin; Pres, pressure.
