# Supplementary material for: Spatiotemporal transitions in Pseudo-nitzschia species assemblages and domoic acid along the Alaska coast
Source: PLoS One. 2023 Mar 22;18(3):e0282794. doi: 10.1371/journal.pone.0282794 (PMC10032537; doi:10.1371/journal.pone.0282794)
Supplement: S6 Table — The ANOSIM test was applied to surveys (Healy 1801 in summer and/or Healy 1803 in fall), subregions (Bering Strait, Chukchi Sea, Barrow Canyon, Beaufort Sea) and water masses. Significant level of sample statistic is 0.1% (marked in bold). (DOCX) [file pone.0282794.s008.docx]

**S6 Table. Results for the ANOSIM permutation-based hypothesis testing.** The ANOSIM test was applied to surveys (*Healy* 1801 in summer and/or *Healy* 1803 in fall), subregions (Bering Strait, Chukchi Sea, Barrow Canyon, Beaufort Sea) and water masses. Significant level of sample statistic is 0.1% (marked in bold).

| **Group by cruise(s)** | **R statistic** | **p-value** |
| --- | --- | --- |
| ***Healy* 1801 - summer** |  |  |
| Bering, Chukchi | -0.234 | p>0.05 |
| Bering, Barrow | -0.189 | p>0.05 |
| Chukchi, Barrow | -0.088 | p>0.05 |
| ***Healy* 1803 - fall** |  |  |
| Chukchi, Beaufort | 0.322 | **p<0.001** |
| Chukchi, Barrow | 0.731 | **p<0.001** |
| Beaufort, Barrow | 0.275 | **p<0.001** |
| ***Healy* 1801 & 1803 - summer and fall** | | |
| Bering, Chukchi | 0.214 | p>0.05 |
| Bering, Barrow | -0.144 | p>0.01 |
| Bering, Beaufort | 0.41 | **p<0.001** |
| Chukchi, Barrow | 0.041 | **p<0.05** |
| Chukchi, Beaufort | 0.656 | **p<0.001** |
| Barrow, Beaufort | 0.605 | **p<0.001** |
| ***Healy* 1801 - summer** |  |  |
| ACW, BSW | 0.278 | **p<0.002** |
| ACW, MWR | 0.192 | **p<0.001** |
| ACW, RWW | 0.629 | **p<0.001** |
| ACW, NVWW | 0.999 | **p<0.05** |
| BSW, MWR | -0.001 | p>0.05 |
| BSW, RWW | 0.28 | **p<0.001** |
| BSW, NVWW | 0.886 | **p<0.05** |
| MWR, RWW | 0.348 | **p<0.001** |
| MWR, NVWW | 0.766 | p>0.05 |
| RWW, NVWW | -0.103 | p>0.05 |
| ***Healy* 1803 - fall** |  |  |
| ACW, MWR | 0.258 | p>0.05 |
| ACW, NVWW | 0.955 | **p<0.001** |
| ACW, RWW | 0.778 | **p<0.05** |
| ACW, BSW | 0.223 | p>0.05 |
| MWR, NVWW | 0.384 | **p<0.001** |
| MWR, RWW | 0.118 | p>0.05 |
| MWR, BSW | 0.165 | **p<0.05** |
| NVWW, RWW | 0.477 | **p<0.001** |
| NVWW, BSW | 0.899 | **p<0.001** |
| RWW, BSW | 0.49 | **p<0.05** |

ACW, Alaskan Coastal Water; BSW, Bering Summer Water; MRW, Melt Water /River Water; NVWW, Newly Ventilated Winter Water; RWW, Remnant Winter Water.
